# Radiotherapy in combination with CD47 blockade elicits a macrophage-mediated abscopal effect

**DOI:** 10.1038/s43018-022-00456-0

**Published:** 2022-11-21

**Authors:** Yoko Nishiga, Alexandros P. Drainas, Maya Baron, Debadrita Bhattacharya, Amira A. Barkal, Yasaman Ahrari, Rebecca Mancusi, Jason B. Ross, Nobuyuki Takahashi, Anish Thomas, Maximilian Diehn, Irving L. Weissman, Edward E. Graves, Julien Sage

**Affiliations:** 1 Department of Radiation Oncology, Stanford University, Stanford, CA USA; 2 Department of Pediatrics, Stanford University, Stanford, CA USA; 3 Department of Genetics, Stanford University, Stanford, CA USA; 4 Institute for Stem Cell Biology and Regenerative Medicine, Stanford University, Stanford, CA USA; 5 Ludwig Center for Cancer Stem Cell Research and Medicine, Stanford University, Stanford, CA USA; 6 Department of Pathology, Stanford University, Stanford, CA USA; 7 Developmental Therapeutics Branch, Center for Cancer Research, National Cancer Institute, Bethesda, MD USA; 8 Department of Medical Oncology, National Cancer Center Hospital East, Kashiwa, Japan

**Keywords:** Tumour immunology, Small-cell lung cancer, Cancer

## Abstract

Radiation therapy is a mainstay of cancer treatment but does not always lead to complete tumor regression. Here we combine radiotherapy with blockade of the ‘don’t-eat-me’ cell-surface molecule CD47 in small cell lung cancer (SCLC), a highly metastatic form of lung cancer. CD47 blockade potently enhances the local antitumor effects of radiotherapy in preclinical models of SCLC. Notably, CD47 blockade also stimulates off-target ‘abscopal’ effects inhibiting non-irradiated SCLC tumors in mice receiving radiation. These abscopal effects are independent of T cells but require macrophages that migrate into non-irradiated tumor sites in response to inflammatory signals produced by radiation and are locally activated by CD47 blockade to phagocytose cancer cells. Similar abscopal antitumor effects were observed in other cancer models treated with radiation and CD47 blockade. The systemic activation of antitumor macrophages following radiotherapy and CD47 blockade may be particularly important in patients with cancer who suffer from metastatic disease.

## Abstract (web summary)

Nishiga and colleagues show therapeutic efficacy for a combination of radiotherapy and CD47 blockade, which also elicits an abscopal effect mediated by macrophages migrating into non-irradiated tumor sites and phagocytosing cancer cells.

## Main

While targeted therapies have been successfully developed against a number of cancer types, radiation therapy (radiotherapy) remains a mainstay of cancer treatment, with more than 50% of all patients with cancer receiving radiotherapy during the course of their disease^1^. The primary mode of action of radiotherapy is the direct induction of cancer cell death through acute damage to DNA^2^. Recent technological advances, including intensity-modulated radiation therapy and image-guided radiation therapy, have facilitated both dose escalation to tumors and dose sparing to surrounding normal tissues. As a result, radiotherapy is now applied effectively and safely for a growing number of patients^3^. Unfortunately, however, radiotherapy does not always lead to complete tumor elimination and its role as a focal therapy limits its use in treating metastatic disease.

These clinical observations have led to a search for strategies combining radiotherapy and other therapies. In particular, there is growing interest in combining radiotherapy with immunotherapies, most commonly strategies promoting the systemic activation of T cells^4,5^. In this context, the release of tumor antigens by dead or dying irradiated cancer cells is thought to be a mechanism that can enhance the priming of antigen-specific T cells, thereby activating an adaptive immune response against any remaining cancer cells^6,7^. This idea has led to the initiation of a number of clinical trials^8,9^; however, radiotherapy can activate both innate and adaptive immune responses, and these responses can be pro- or antitumor growth^6,7^. Therefore, it is important to continue to investigate combination therapies that include immune cell types beyond T cells.

SCLC represents ~15% of all lung cancers and causes over 200,000 deaths worldwide each year. SCLC is heavily linked to tobacco smoking. Unfortunately, the number of SCLC-related deaths continues to rise worldwide, along with increasing numbers of smokers^10,11^. A major clinical challenge is that ~65% of patients with SCLC have metastatic disease at the time of diagnosis; these patients have a 5-year survival rate of 1–2%^12^. Even patients with localized disease at diagnosis have dismal survival rates of generally less than 2 years. In patients with SCLC, radiotherapy is often combined with chemotherapy and is widely used with both curative and palliative intent^13,14^. Patients with SCLC with localized disease have been managed with first-line radiotherapy combined with chemotherapy for decades. Patients with SCLC usually respond well initially, but most patients then relapse rapidly^14^. Recently, immunotherapies focusing on the inhibition of PD-1/PD-L1 and subsequent activation of T cells, in combination with standard of care, have resulted in improved overall survival rates in patients with SCLC^15,16^; however, overall outcomes for this tumor type remain limited and there remains a great unmet need to develop more effective therapeutic approaches enhancing the activity of T cells or other immune cells^17^.

The CD47 cell surface molecule serves as a myeloid checkpoint: blockade of the interactions between CD47 expressed by cancer cells and SIRPα on the surface of macrophages alleviates a ‘don’t-eat-me’ signal, facilitating the phagocytosis of cancer cells by macrophages^18,19^. We previously found that CD47 is highly expressed on the surface of SCLC cells and that blockade of CD47 can enhance the phagocytosis of SCLC cells by macrophages^20^. Here we asked whether combining CD47 blockade and radiotherapy could synergize to inhibit the growth of SCLC tumors in preclinical models of SCLC. We made the surprising observation that CD47 blockade not only potentiates local antitumor effects of radiotherapy but also stimulates abscopal effects, inhibiting the growth of distant, non-irradiated tumors. Based on these observations, we systematically deconstructed the cellular mechanisms underlying local and abscopal responses to radiotherapy and CD47 blockade.

## Results

### CD47 blockade enhances the anti-SCLC effects of radiotherapy

**Fig. 1:**
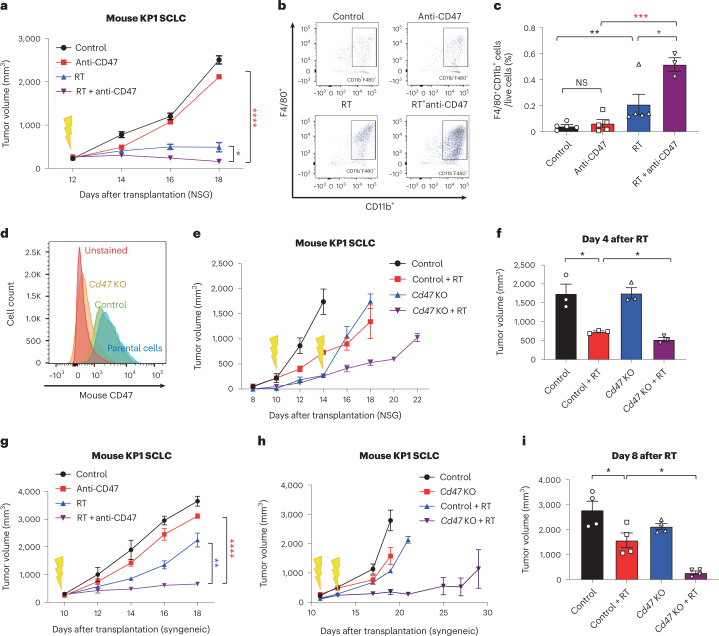
CD47 blockade enhances local tumor inhibition following irradiation of SCLC tumors. **a**, Growth curves of KP1 SCLC allografts in immunodeficient NSG mice with the indicated treatments. *n* = 4 (RT + anti-CD47) or *n* = 5 (control, anti-CD47 and RT) mice. ****P < 0.0001, *P = 0.0159. **b**, Tumor-infiltrating macrophages (CD11b^+^F4/80^+^) identified by flow cytometry from tumors collected in **a**. **c**, Quantification of tumor-infiltrating macrophages from **b**. Data are representative of *n* = 2 independent experiments. *n* = 3 (RT + anti-CD47) or *n* = 5 (control, anti-CD47 and RT) tumors. **P = 0.0079, *P = 0.0320, ****P = 0.0002. **d**, CD47 expression for KP1 or KP1 *Cd47* knockout cells by flow cytometry. **e**, Growth curves of KP1 control and *Cd47* knockout allografts in NSG mice. **f**, Quantification of tumor volume 4 d after radiation. *n* = 1 experiment with *n* = 3 tumors per condition. *P = 0.0170, *P = 0.0279. **g**, Growth curves of KP1 SCLC allografts in B6129SF1 immunocompetent recipient mice with the indicated treatments. *n* = 1 experiment with *n* = 3 (RT + anti-CD47) or *n* = 4 (control, anti-CD47 and RT) tumors. **h**, Growth curves of KP1 control and *Cd47* knockout SCLC allografts in immunocompetent syngeneic mice. Tumors were irradiated at two different time points to account for the slower growth of *Cd47* knockout cells. **i**. Quantification of tumor volume 8 d after radiation. *n* = 1 experiment with *n* = 4 tumors. *P = 0.0286, *P = 0.0390. Two-tailed Student’s *t*-tests following two-way analysis of variance (ANOVA) were performed in **a** (*P* < 0.0001) and **g** (*P* < 0.0001). Two-tailed Student’s *t*-tests following one-way ANOVA were performed in **c** (*P* = 0.001), **f** (*P* = 0.0006) and **i** (*P* < 0.0001). Error bars represent s.e.m. *P < 0.05, **P < 0.01, ***P < 0.001, ****P < 0.0001. Source data

**Extended Data Fig. 1:**
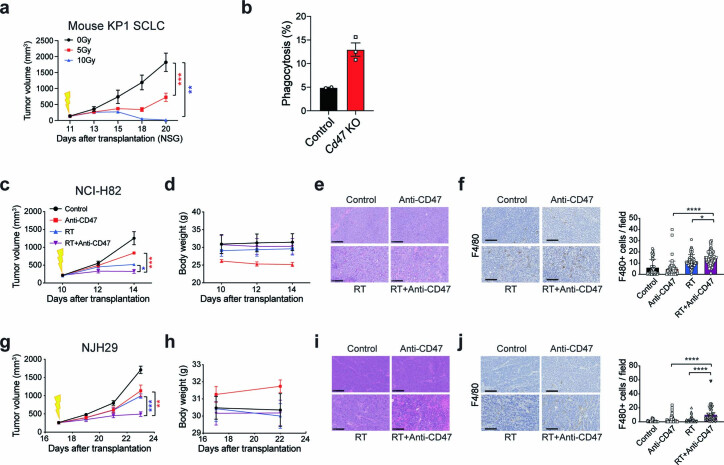
CD47 blockade enhances local antitumor effects following radiation in murine and human SCLC models. **a**. Growth curves of KP1 SCLC allografts in NSG mice irradiated with 0, 5, or 10 Gy. N = 1 experiment with n = 5 tumors for each condition. ***p = 0.003, **p = 0.0079. b. In vitro phagocytosis assay performed with mouse bone marrow-derived macrophages (BMDMs) and KP1 mouse SCLC control and *Cd47* knockout cells. N = 1 experiment with triplicates of the primary cultures. **c**–**f**. Experiments with NCI-H82 SCLC xenografts in NSG mice. c. Growth curves with the indicated treatments. N = 1 experiment with n = 4 mice. ***p = 0.0002, *p = 0.0225. **d**. Body weight of mice. **e**. Representative H&E (hematoxylin and eosin) of NCI-H82 tumor sections. **f**. Histological analysis of macrophage infiltration in SCLC xenografts. Specimens were stained for the macrophage marker, F4/80 (left) and the signal was quantified (right). Scale bar, 100 µm. N = 1 experiment with n = 4 tumors. ****p < 0.0001, *p = 0.0136. **g–j**. Same as (**c**–**f**) for NJH29 SCLC xenografts. N = 1 experiment with n = 4 (Control, RT) or n = 5 (Anti-CD47, RT + Anti-CD47) mice. g. **p = 0.0054, ***p = 0.0009. j. ****p < 0.0001. Two-tailed unpaired t-tests were performed in (b) with primary BMDMs. Two-tailed t-tests following two-way ANOVA were performed in (**a**) (p < 0.0001), (**c**) (p = 0.0004) and (**g**) (p = 0.0007). Two-tailed t-tests following one-way ANOVA were performed in (**f**) (p < 0.0001) and (**j**) (p < 0.0001). Error bars represent SEM. *p < 0.05, **p < 0.01, ***p < 0.001, ****p < 0.0001. Source data

Based on the frequent use of radiation in patients with SCLC and our previous findings with CD47 blockade in preclinical models of SCLC^20^, we evaluated whether irradiation of SCLC tumors could be combined with CD47 blockade to improve antitumor responses. We initially engrafted mouse SCLC cell lines into the flanks of NSG mice, which lack functional T cells, B cells and natural killer (NK) cells but retain functional monocytes and macrophages. In this context, we found that 5 Gy irradiation inhibited tumor growth and 10 Gy almost eradicated the tumors (Extended Data Fig. 1a). Based on this observation, we applied a single-fraction dose of 5 Gy in subsequent experiments to be able to identify additional antitumor effects of combination therapies. As a single-agent treatment, CD47-blocking antibody had minimal effects on the growth of tumors (Fig. 1a), possibly due to cross-reactivity to CD47 expressed on red blood cells and other cells in the body, with fewer molecules of antibody being able to bind to mouse SCLC cells^21^. In contrast, the combination treatment with irradiation and CD47 blockade significantly inhibited tumor growth (Fig. 1a). Enhanced tumor inhibition in the combination treatment correlated with a further increase in the infiltration of macrophages to the tumor microenvironment relative to either treatment alone (Fig. 1b,c). Similarly, *Cd47* knockout SCLC cells were more efficiently phagocytosed by primary macrophages in culture, as expected (Fig. 1d and Extended Data Fig. 1b) and formed smaller tumors, which was further enhanced by radiation therapy (Fig. 1e,f). Irradiation combined with a blocking antibody directed against human CD47 also enhanced antitumor effects with two human SCLC xenograft models, with increased macrophage infiltration and no significant body weight loss (Extended Data Fig. 1c–j). Finally, pharmacologic or genetic CD47 inhibition in subcutaneous mouse tumors growing in immunocompetent mice still led to enhanced tumor inhibition following irradiation, indicating that the antitumor effects observed are not adversely affected by the presence of T cells, B cells or NK cells (Fig. 1g–i). Thus, CD47 inhibition enhances the antitumor effects of local radiotherapy in murine and human preclinical models of SCLC.

### CD47 blockade promotes abscopal effects in SCLC

Clinical case reports have described so-called ‘abscopal effects’ of radiation therapy, where irradiation of a tumor mass results in antitumor effects on another non-irradiated tumor lesion in the same individual^22^. The mechanisms mediating these abscopal effects remain poorly understood but are commonly thought to involve the adaptive immune system through tumor-associated antigen cross-priming and activation of cytotoxic CD8^+^ T cells^23–25^. Because CD47 blockade has also been associated with activation of antitumor T cells in some contexts^26–28^, we sought to evaluate whether inhibition of CD47 could facilitate abscopal responses to irradiation.

**Fig. 2:**
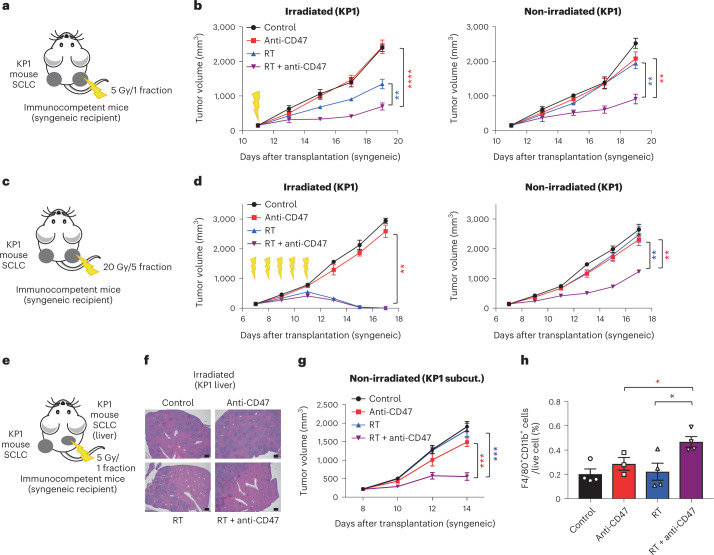
The combination of radiotherapy and CD47 blockade leads to abscopal effects in preclinical mouse models of SCLC. **a**, Mouse KP1 SCLC cells were engrafted into both flanks of B6129SF1 immunocompetent syngeneic mice and only right-side tumors were irradiated. **b**, Growth curves of KP1 SCLC allografts as in **a** with the indicated treatments (irradiated tumors on left, non-irradiated tumors on right). *n* = 1 experiment with *n* = 4 (RT) or *n* = 5 (control, anti-CD47 and RT + anti-CD47) mice. Irradiated tumors, ****P < 0.0001, **P = 0.0061; non-irradiated tumors, **P = 0.0012, **P = 0.0020. **c**, Mouse KP1 SCLC cells were engrafted into both flanks of B6129SF1 immunocompetent syngeneic mice. Right-side tumors received 20 Gy in five fractions. **d**, Growth curves of KP1 SCLC allografts with the indicated treatments in irradiated and non-irradiated control tumors. *n* = 1 experiment with *n* = 5 mice (two tumors per mouse). Irradiated tumors, **P = 0.0079; non-irradiated tumors, **P = 0.0079, **P = 0.0079. **e**, KP1 cells were both intravenously injected and engrafted into the right side of flank of B6129SF1 mice. The cells that were injected intravenously formed liver metastases. Only these liver metastases were irradiated. **f**, Representative image of liver sections stained with hematoxylin and eosin (H&E). Scale bar, 500 µm. *n* = 1 experiment with *n* = 5 mice. **g**, Growth curves of non-irradiated KP1 SCLC subcutaneous (subcut.) allografts. *n* = 1 experiment with *n* = 5 mice. ***P = 0.0003, ***P = 0.0003. **h**, Quantification of tumor-infiltrating macrophages (CD11b^+^F4/80^+^) identified by flow cytometry from subcutaneous non-irradiated tumors collected in **e**. *n* = 1 experiment with *n* = 3 (anti-CD47) or *n* = 4 (control, RT and RT + anti-CD47) tumors. *P = 0.0440, *P = 0.0251. Two-tailed Student’s *t*-tests following two-way ANOVA were performed in **b** (irradiated tumors, *P* < 0.0001; non-irradiated tumors, *P* = 0.0003), **d** (irradiated tumors, *P* < 0.0001; non-irradiated tumors, *P* < 0.0001) and **g** (*P* < 0.0001). Two-tailed Student’s *t*-tests following one-way ANOVA were performed in **h** (*P* = 0.016). Error bars represent s.e.m. *P < 0.05, **P < 0.01, ***P < 0.001, ****P < 0.0001. Source data

We first engrafted mouse SCLC cells into both flanks of immunocompetent recipient mice and allowed tumors to grow for approximately 2 weeks. Radiotherapy was delivered focally to only the tumor on the right side of each mouse, with or without concurrent systemic anti-CD47 antibody treatment (Fig. 2a). Similar to mice with a single tumor, radiotherapy and CD47 blockade had an enhanced local inhibitory effect on the irradiated tumors compared to individual treatments (Fig. 2b, left). Notably, mice treated with the combination therapy had significantly smaller non-irradiated tumors compared to mice given either treatment alone (Fig. 2b, right). Similar results were observed using a fractionated radiotherapy schedule (20 Gy in five fractions) that more closely resembles the regimen that patients with SCLC undergo in the clinic and that almost completely eliminated the irradiated tumor (Fig. 2c,d). This effect was also observed when we irradiated liver metastases and measured the growth of subcutaneous SCLC tumors in the same mice (Fig. 2e–h). In all of these settings, using different doses of radiation and tumors at different sites, the abscopal effect was only visible following the combination therapy.

### Abscopal responses in SCLC are independent of T cells

**Fig. 3:**
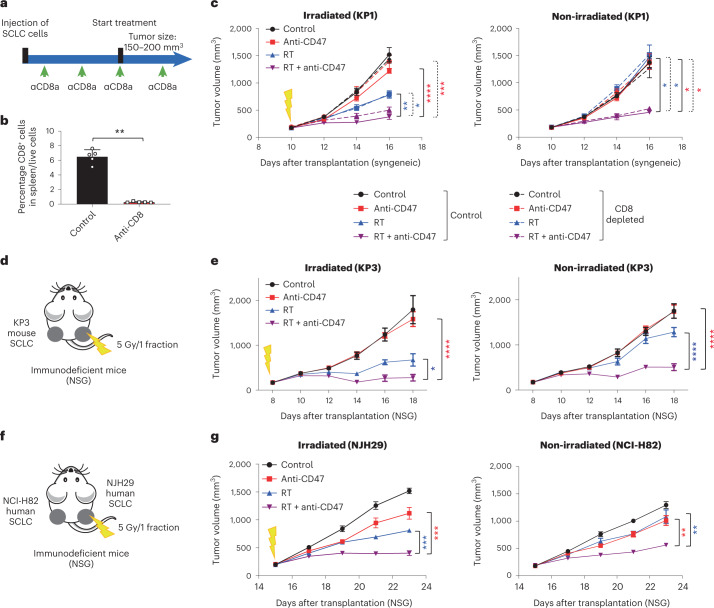
The abscopal effects induced by the combination of radiotherapy and CD47 blockade are independent of T cells. **a**, Mouse KP1 SCLC cells were engrafted into both flanks of B6129SF1 immunocompetent syngeneic mice and only right-side tumors were irradiated. Schematic of the depletion of CD8^+^ T cells following anti-CD8 antibody treatment. **b**, Analysis of splenic T cells by flow cytometry as in **c**. *n* = 1 experiment with *n* = 5 tumors. **P = 0.0079. **c**, Growth curves of KP1 SCLC allografts with the indicated treatments. *n* = 1 experiment with *n* = 5 mice except RT + anti-CD47/control and RT + anti-CD47/CD8 depletion (*n* = 4 mice). See independent experiment in Extended Data Fig. 2a. Irradiated tumors, ****P < 0.0001; ***P = 0.007, **P = 0.0016, *P = 0.0109; non-irradiated tumors, *P = 0.0159, *P = 0.0159, *P = 0.0159, *P = 0.0159. **d**, As in **a** with mouse KP3 cells engrafted into both flanks of NSG immunodeficient mice. **e**, Growth curves of KP3 allografts as in **d** with the indicated treatments. *n* = 1 experiment with *n* = 6 (control, anti-CD47 and RT) or *n* = 7 (RT + anti-CD47) tumors. Irradiated tumors, ****P < 0.0001, *P = 0.0229; non-irradiated tumors, ****P < 0.0001. **f**, As in **a** with human NCI-H82 and NJH29 SCLC cells were engrafted into NSG immunocompromised mice and only NJH29 tumors were irradiated. **g**, Growth curves of SCLC xenografts as in **f** with the indicated treatments. *n* = 1 experiment with *n* = 5 mice. Irradiated tumors, ***P = 0.0002, ***P = 0.0001; non-irradiated tumors, **P = 0.0079, **P = 0.0079. Two-tailed unpaired Student’s *t*-tests were performed in **b**. Two-tailed Student’s *t*-tests following two-way ANOVA were performed in **c** (irradiated tumors, *P* < 0.0001; non-irradiated tumors, *P* < 0.0001), **e** (irradiated tumors, *P* < 0.0001; non-irradiated tumors, *P* < 0.0001) and **g** (irradiated tumors, *P* < 0.0001; non-irradiated tumors, *P* < 0.0001). Error bars represent s.e.m. *P < 0.05, **P < 0.01, ***P < 0.001, ****P < 0.0001. Source data

**Extended Data Fig. 2:**
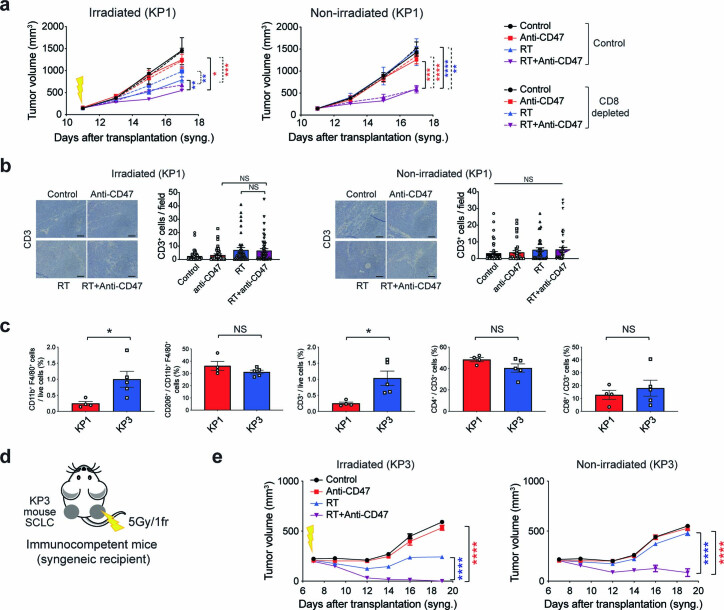
Abscopal effects upon radiation therapy and CD47 blockade in the KP1 mouse model of SCLC are independent of T cells. **a**. Growth curves of KP1 SCLC allografts with the indicated treatments in irradiated and non-irradiated tumors. N = 1 experiment with n = 5 mice. Independent experiment shown in Fig. 3c. Irradiated tumors: *p = 0.0159, ***p = 0.0001, **p = 0.0079, **p = 0.0023, non-irradiated tumors: ***p = 0.0003, ****p < 0.0001, **p = 0.0079. **b**. Histological analysis and quantification of T-cell infiltration as in Fig. 3c by immunostaining for CD3. Sections were counterstained with hematoxylin. Each symbol represents one field quantified. Scale bar, 100 µm. N = 1 experiment with n = 5 mice except RT + anti-CD47/Control and RT + Anti-CD47/CD8 depletion (n = 4 mice). **c**. Populations of macrophages and T cells (antibodies indicated) quantified by flow cytometry in the KP1 and KP3 mouse allograft models in B6129SF1 hosts. N = 1 experiment with n = 4 (KP1) or n = 5 (KP3) mice. *p = 0.0317, *p = 0.0159. **d**. KP3 SCLC cells were engrafted into both flanks of B6129SF1 immunocompetent syngeneic mice and only right-side tumors were irradiated. **e**. Growth curves of KP3 allografts with the indicated treatments in irradiated and non-irradiated control tumors. N = 1 experiment with n = 7 (Anti-CD47) or n = 8 (Control), or n = 9 (RT and RT + Anti-CD47) mice (2 tumors per mouse). 5/9 mice had complete remission at both sides in the combination treatment arm. ****p < 0.0001. Two-tailed t-tests were performed in (**c**). Two-tailed t-tests following one-way ANOVA were performed in (**b**) (irradiated tumors: p = 0.0054, non-irradiated tumors: p = 0.30). Two-tailed t-tests following two-way ANOVA were performed in (**a**) (irradiated tumors: p < 0.0001, non-irradiated tumors: p < 0.0001) and (**e**) (irradiated tumors: p < 0.0001, non-irradiated tumors: p < 0.0001). Error bars represent SEM. *p < 0.05, **p < 0.01, ***p < 0.001, ****p < 0.0001. Source data

Based on the commonly accepted model of T cells mediating abscopal responses, we expected that the abscopal effects observed in this model with radiotherapy and CD47 blockade would be decreased or eliminated in the absence of active cytotoxic T cells; however, when we depleted CD8^+^ T cells by in vivo treatment with anti-CD8 antibodies (Fig. 3a,b), this depletion did not prevent stimulation of an abscopal effect in mice treated with the combination therapy (Fig. 3c and Extended Data Fig. 2a). Accordingly, infiltration of T cells was not increased by the combination therapy compared to either treatment alone (Extended Data Fig. 2b). A second mouse SCLC allograft model with more prominent T-cell infiltration (KP3) responded similarly to radiotherapy and CD47 blockade in immunocompetent syngeneic hosts and T-cell-deficient NSG hosts (Fig. 3d,e and Extended Data Fig. 2c–e), further indicating that T cells are not required for the observed abscopal effects in the context of CD47 blockade.

**Extended Data Fig. 3:**
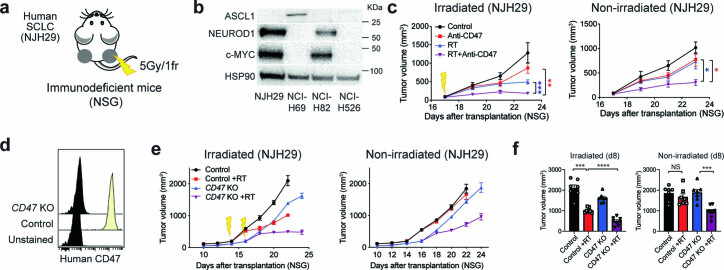
T-cell independent abscopal effects upon radiation therapy and CD47 blockade in the NJH29 SCLC model. **a**. Human SCLC cells (NJH29) were engrafted into both flanks of NSG mice and only right-side tumors were irradiated. **b**. Immunoblot analysis for ASCL1, NEUROD1, c-MYC. HSP90 is a loading control. N = 1 experiment. **c**, **e**. Growth curves of xenografts with the indicated treatments. **c**. NJH29: N = 1 experiment with n = 5 mice per condition. Irradiated tumors: **p = 0.0022, ***p = 0.0008, non-irradiated tumors: *p = 0.0113, *p = 0.0241. **d**. CD47 expression by flow cytometry in NJH29 control and CD47 knockouts. **e**. NJH29: N = 1 experiment with n = 5 mice. NSG mice were engrafted with either two control tumors or two knockout tumors. **f**. Quantification of tumor volume 8 days after radiation in irradiated and non-irradiated control tumors in (**e**). N = 1 experiment with n = 5 mice. Irradiated tumors: ***p = 0.0006, ****p < 0.0001, non-irradiated tumors: ***p = 0.0002. Two-tailed t-tests following two-way ANOVA were performed in (**c**) (irradiated tumors: p = 0.0015, non-irradiated tumors: p = 0.0055). Two-tailed t-tests following one-way ANOVA were performed in (**f**) (irradiated tumors: p < 0.0001, non-irradiated tumors: p = 0.0001). Source data

**Extended Data Fig. 4:**
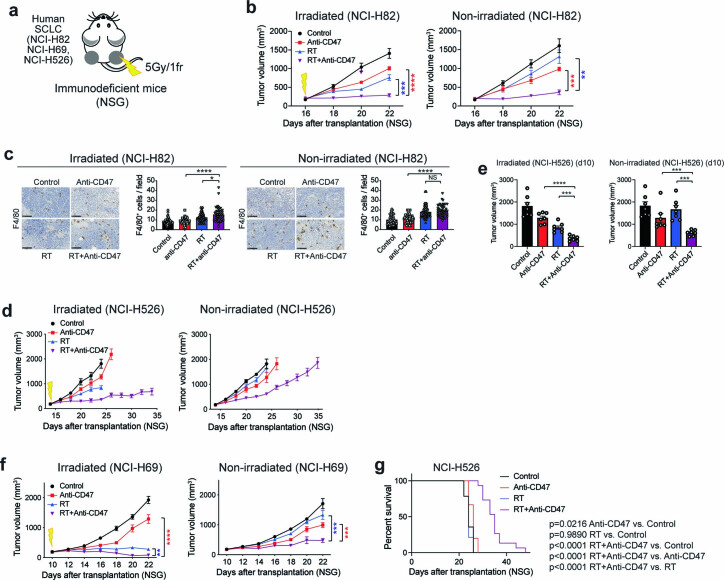
T-cell independent abscopal effects upon radiation therapy and CD47 blockade in human SCLC models. **a**. Human SCLC cells were engrafted into both flanks of NSG mice and only right-side tumors were irradiated. **b**, **d**, **f**, Growth curves of xenografts with the indicated treatments. b. NCI-H82: N = 1 experiment with n = 4 (Control, Anti-CD47) or 5 (RT, RT + Anti-CD47) mice. Irradiated tumors: ****p < 0.0001, ***p = 0.0007, non-irradiated tumors: ***p = 0.0002, **p = 0.0010. c. Histological analysis and quantification of macrophages tumors as in (**b**) by immunostaining for F4/80. Sections were counterstained with hematoxylin. Each symbol represents one field quantified. Scale bar, 100 µm. Irradiated tumors: *p = 0.0282, ****p < 0.0001, non-irradiated tumors: ****p < 0.0001. **d**. NCI-H526: N = 1 experiment with n = 7 mice per condition. e. Quantification of tumor volume 10 days after radiation in irradiated and non-irradiated control tumors in (**d**). N = 1 experiment with n = 7 mice per condition. Irradiated tumors: ****p < 0.0001, ***p = 0.0002, non-irradiated tumors: ***0.0006, ***p = 0.0006. **f**. NCI-H69: N = 1 experiment with n = 7 (Control, Anti-CD47, RT) or 8 (RT + Anti-CD47) mice per condition. Irradiated tumors: ****p < 0.0001, **p = 0.0064, non-irradiated tumors: ***p = 0.0004, ***p = 0.0002. **g**. Survival curves of NCI-H526 xenografts with the indicated treatments: N = 1 experiment with n = 14 (Control, RT) or n = 15 (Anti-CD47, RT + Anti-CD47) mice. Two-tailed t-tests following two-way ANOVA were performed in (**b**) (irradiated tumors: p < 0.0001, non-irradiated tumors: p < 0.0001), and (**f**) (irradiated tumors: p < 0.0001, non-irradiated tumors: p < 0.0001). Two-tailed t-tests following one-way ANOVA were performed in (**c**) (irradiated tumors: p < 0.0001, non-irradiated tumors: p < 0.0001) and (**d**) (irradiated tumors: p < 0.0001, non-irradiated tumors: p = 0.0002). Survival curves were compared using the Log-rank test in (**g**). Error bars represent SEM. *p < 0.05, **p < 0.01, ***p < 0.001, ****p < 0.0001. Source data

To further test whether abscopal effects observed in the combination therapy are independent of T cells, we performed experiments with xenograft models in NSG mice. We used four human SCLC cell lines (NJH29, NCI-H82, NCI-H69 and NCI-H526) representing different subtypes of SCLC. The induction of abscopal responses by radiotherapy combined with CD47 blockade or *CD47* knockout was observed for all of these models, along with increased recruitment of macrophages to both the irradiated and non-irradiated sites (Extended Data Figs. 3a–f and 4a–f). Radiotherapy combined with CD47 blockade also produced significant survival benefits in the NCI-H526 xenograft model (Extended Data Fig. 4g). Moreover, when mice were transplanted with NJH29 cells in the right flank and NCI-H82 cells in the left flank, representing two models derived decades apart from two patients with SCLC, irradiation of NJH29 xenografts also led to the inhibition of NCI-H82 xenografts on the contralateral non-irradiated side in the combination therapy group (Fig. 3f,g). These results further indicate that the antitumor abscopal effects observed upon irradiation and CD47 blockade are not due to an adaptive immune system response against specific tumor antigens.

### Macrophages are required for abscopal responses in SCLC

**Fig. 4:**
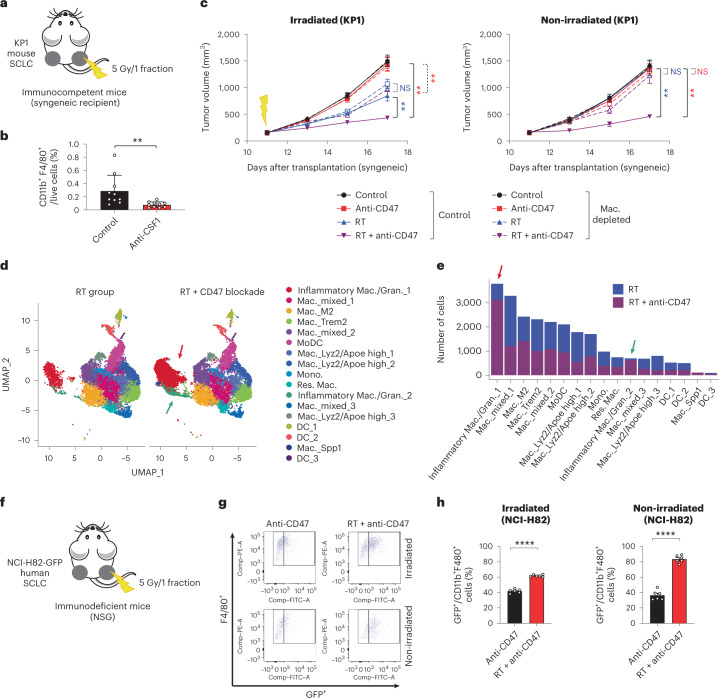
Inflammatory macrophages mediate abscopal effects induced by radiotherapy and CD47 blockade. **a**, Mouse KP1 SCLC cells were engrafted into both flanks of B6129SF1 immunocompetent syngeneic mice and only right-side tumors were irradiated. **b**, Macrophages were depleted using an anti-CSF1 antibody, as quantified by flow cytometry (CD11b^+^F4/80^+^ cells) from the tumors of mice in the control group with or without anti-CSF1 antibody on day 17. *n* = 1 experiment with *n* = 5 mice (two tumors per mouse), *P* = 0.0010. **c**, Growth curves of KP1 SCLC allografts as in **a**,**b** with the indicated treatments. *n* = 1 experiment with *n* = 5 mice (two tumors per mouse). Irradiated tumors, **P = 0.0079, **P = 0.0011, **P = 0.0038; non-irradiated tumors, **P = 0.0079, **P = 0.0079. NS, not significant; Mac., macrophages. **d**, Uniform Manifold Approximation and Projection (UMAP) dimension 1 and 2 plots of viable CD45^+^ cells in non-irradiated KP1 tumors in NSG mice in the RT and RT/CD47-blockade treatment groups. Cell clusters are colored by cell populations. Colored arrows point to two groups of inflammatory macrophages whose numbers increase in non-irradiated tumors in the RT/CD47-blockade treatment group. **e**, Number of cells in each subpopulation identified in the scRNA-seq analysis in the two treatment groups. **f**, Human NCI-H82 cells stably expressing green fluorescent protein (GFP) were engrafted into both flanks of NSG mice and only right-side tumors were irradiated. **g**, Example of a flow cytometry analysis of CD11b^+^F4/80^+^ macrophages also positive for GFP (indicative of phagocytosis) in the two treatments were irradiated. Schematic of the depletion on CD11b^+^ cells. **h**, Quantification of **g**. Phagocytosis was measured 6 d after treatment start as the percentage of CD11b^+^F4/80^+^ macrophages that are also GFP^+^. *n* = 1 experiment with *n* = 6 mice. ****P < 0.0001. Two-tailed Student’s *t*-tests following two-way ANOVA were performed in **c** (irradiated tumors, *P* < 0.0001; non-irradiated tumors, *P* < 0.0001). Two-tailed Student’s *t*-tests were performed in **b** and **h**. Error bars represent s.e.m. *P < 0.05, **P < 0.01, ***P < 0.001, ****P < 0.0001. Source data

**Extended Data Fig. 5:**
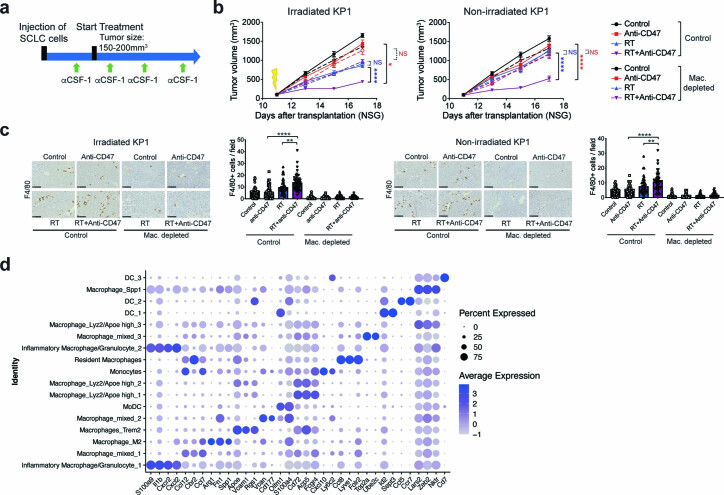
Abscopal effects of combination of radiation and CD47 blockade are mediated by macrophages in SCLC. **a** Macrophages were depleted with anti-CSF1 antibody treatment as indicated. **b**. Mouse KP1 SCLC cells were engrafted into both flanks of NSG mice and only right-side tumors were irradiated. Growth curves of KP1 SCLC allografts with the indicated treatments. N = 1 experiment with n = 5 tumors. Irradiated tumors: *p = 0.0398, ****p < 0.0001, non-irradiated tumors: ****p < 0.0001. **c**. Histological analysis and quantification of macrophages infiltrating irradiated and non-irradiated tumors from Fig. 4c by immunostaining for F4/80. Sections were counterstained with hematoxylin. Each symbol represents one field quantified. Scale bar, 100 µm. N = 1 experiment with n = 5 mice. Irradiated tumors: ****p < 0.0001, **p = 0.0033, non-irradiated tumors: ****p < 0.0001, **p = 0.0045. **d**. Expression of representative genes for subpopulations of CD45 + hematopoietic cells in SCLC tumors from the scRNA-seq analysis as in Fig. 4d. The % of cells expressing the genes is indicated by the size of the circle. Two-tailed t-tests following one-way ANOVA were performed in (**c**) (irradiated tumors: p < 0.0001, non-irradiated tumors: p < 0.0001). Two-tailed t-tests following two-way ANOVA were performed in (**b**) (irradiated tumors: p < 0.0001, non-irradiated tumors: p < 0.0001). Error bars represent SEM. *p < 0.05, **p < 0.01, ***p < 0.001, ****p < 0.0001. Source data

The observation that T cells are not required for abscopal effects upon irradiation and CD47 blockade raised the question of the cellular mechanisms underlying these effects. We focused on SCLC models to answer this question. Because the main consequence of CD47 blockade is activation of phagocytosis by macrophages, we evaluated whether macrophages themselves were required. Indeed, we found that depletion of macrophages by in vivo treatment with anti-CSF1 antibodies led to an abrogation of abscopal effects in both immunocompetent and immunodeficient mice bearing SCLC allografts (Fig. 4a–c and Extended Data Fig. 5a–c).

**Extended Data Fig. 6:**
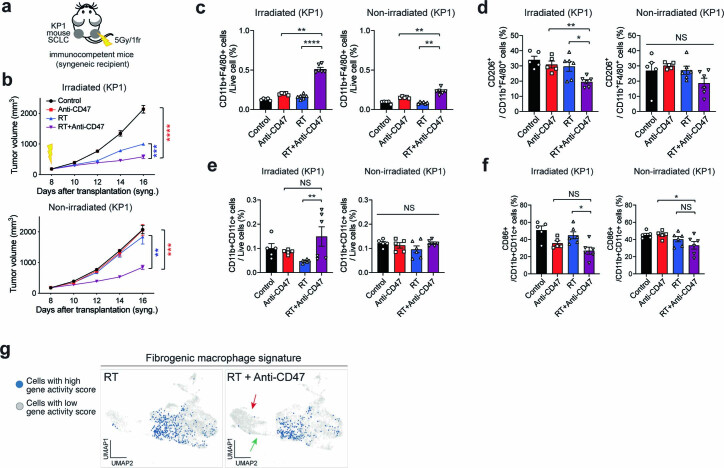
Abscopal effects of combination of radiation and CD47 blockade are associated with tumor-infiltrating macrophages. **a**. Mouse KP1 SCLC cells were engrafted into both flanks of B6129SF1 immunocompetent syngeneic mice and only right-side tumors were irradiated. **b**. Growth curves are shown for the indicated treatments in irradiated and non-irradiated control tumors. N = 1 experiment with n = 5 (Control and Anti-CD47) or n = 6 (RT and RT + Anti-CD47) mice. Irradiated tumors: ****p < 0.0001, ***p = 0.0003, non-irradiated tumors: ***p = 0.0001, **p = 0.0023. **c–f**, Quantification of tumor-infiltrating macrophages (**c**), M2-like macrophages (**d**), dendritic cells (**e**), and CD86 + activated dendritic cells (**f**) in irradiated and non-irradiated tumors from (**a**) using the indicated markers from flow analysis. N = 1 experiment with n = 5 (Control and Anti-CD47) or 6 (RT and RT + Anti-CD47) mice. **c**, irradiated tumors: **p = 0.0043, ****p < 0.0001, non-irradiated tumors: **p = 0.0043, **p = 0.0022, **d**, irradiated tumors: **p = 0.0017, *p = 0.0148, **e**, irradiated tumors: **p = 0.0043, **f**, irradiated tumors: *p = 0.0115, non-irradiated tumors: *p = 0.0412. **g**. Representation of a gene signature associated with fibrosis and SCLC relapse in macrophages in the scRNA-seq dataset. Two-tailed t-tests following two-way ANOVA were performed in (**b**) (irradiated tumors: p < 0.0001, non-irradiated tumors: p < 0.0001). Two-tailed t-tests following one-way ANOVA were performed in (**c**) (irradiated tumors: p < 0.0001, non-irradiated tumors: p < 0.0001), (**d**) (irradiated tumors: p = 0.0025, non-irradiated tumors: p = 0.1154), (**e**) (irradiated tumors: p = 0.0392, non-irradiated tumors: p = 0.2427), and (**f**) (irradiated tumors: p = 0.0031, non-irradiated tumors: p = 0.0432). Error bars represent SEM. *p < 0.05, **p < 0.01, ***p < 0.001, ****p < 0.0001. Source data

As a first step toward understanding the mechanisms underlying abscopal responses mediated by macrophages upon radiotherapy and CD47 blockade in vivo, we sought to better characterize the populations of macrophages present in unirradiated tumors. To this end, we performed single-cell RNA sequencing (scRNA-seq) of CD45^+^ leukocytes at non-irradiated sites in mice treated either with radiotherapy alone (no abscopal responses observed) or radiotherapy and CD47 blockade (abscopal responses observed) in immunocompromised NSG mice. This analysis identified several populations of monocytes/macrophages and dendritic cells (Fig. 4d,e and Extended Data Fig. 5d). Myelomonocytic cells exist in multiple states^29^ and tumor-associated macrophages in particular exist in a spectrum of phenotypes between M1-like antitumor or M2-like protumor macrophages^30^. We found no differences in the number of M1-like and M2-like macrophages between the two treatment groups, as well as no differences in dendritic cells in the scRNA-seq analysis (Fig. 4d,e), which was confirmed in an independent experiment by flow cytometry (Extended Data Fig. 6a–f). The most striking differences between the two treatment groups was the increase in populations of macrophages with inflammatory features in the radiotherapy and CD47 blockade group compared to radiotherapy alone, including two populations with features of inflammatory macrophages/granulocytes (Fig. 4d,e and Extended Data Fig. 5d). Macrophages expressing these markers of inflammation have been associated with antitumor properties^31,32^. Furthermore, the signature of the inflammatory macrophage populations increased in the combination treatment group was distinct from a population of monocyte/macrophages associated with an immunosuppressive microenvironment and tumor recurrence in SCLC^33^ (Extended Data Fig. 6g). Notably, phagocytosis of GFP-expressing SCLC cells by macrophages was increased in vivo in non-irradiated tumors from mice treated by radiotherapy and CD47 blockade compared to CD47 blockade alone (Fig. 4f–h). These observations suggest a model in which the antitumor effects of irradiation and CD47 blockade are mediated by inflammatory macrophages with phagocytic capacity toward cancer cells.

### Irradiation of SCLC cells activates inflammatory responses

**Fig. 5:**
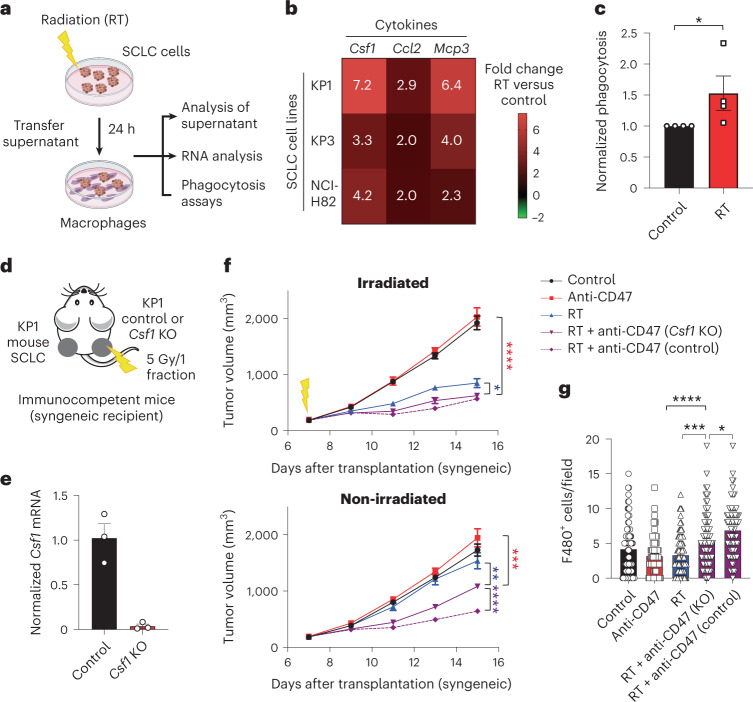
Inflammatory macrophages respond to CSF1 produced by irradiated SCLC cells to mediate abscopal responses. **a**, Schematic of the analysis of the response of SCLC cells to RT in culture. **b**, Heat map of relative mRNA levels for *Csf1*, *Ccl2* and *Mcp3* in irradiated KP1 and KP3 mouse SCLC cells and NCI-H82 human SCLC cells compared to non-irradiated control cells. *n* = 2 independent experiments (average values are shown). **c**, Flow cytometry analysis of in vitro phagocytosis assays with bone-marrow-derived macrophages and KP1 cells fluorescently labeled with calcein AM. *n* = 4 independent experiments shown as the average of technical triplicates. *P = 0.0286. **d**, Mouse KP1 control or *Csf1* knockout (KO) SCLC cells were engrafted into the right flank of B6129SF1 immunocompetent syngeneic mice, with control KP1 cells on the left flank. Only the right side of tumors was irradiated. **e**, Relative mRNA level by quantitative PCR with reverse transcription (RT–qPCR) for *Csf1* in KP1 control and *Csf1* knockout cells. *n* = 3 technical replicates. **f**, Growth curves of KP1 allografts as in **d** at the irradiated and non-irradiated sites. *n* = 1 experiment with *n* = 8 mice. Irradiated tumors, ****P < 0.0001, *P = 0.0289; non-irradiated tumors, ***P = 0.0003, **P = 0.0081, ****P < 0.0001. **g**, Histological quantification of macrophage infiltration in non-irradiated KP1 control and *Csf1* knockout tumors by immunostaining for F4/80 as in **f**. Each symbol represents one field quantified. *n* = 1 experiment with *n* = 7 (anti-CD47) or *n* = 8 (control, RT and RT + anti-CD47 (*Csf1* KO) and RT + anti-CD47 (control)) mice. ****P < 0.0001, ***P = 0.0002, *P = 0.0177. Two-tailed unpaired Student’s *t*-tests were performed in **c**. Two-tailed Student’s *t*-tests following two-way ANOVA were performed in **f** (irradiated tumors, *P* < 0.0001; non-irradiated tumors, *P* < 0.0001). Two-tailed Student’s *t*-tests following one-way ANOVA were performed in **g** (*P* < 0.0001). Error bars represent s.e.m. *P < 0.05, **P < 0.01, ***P < 0.001, ****P < 0.0001. Source data

**Extended Data Fig. 7:**
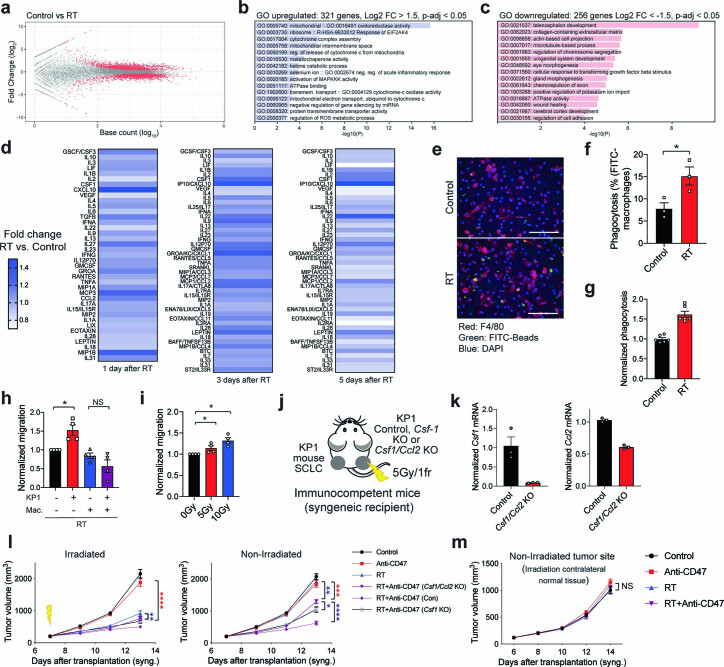
Irradiation of SCLC cells in culture results in the secretion of inflammatory cytokines and enhances the phagocytosis and the migration ability of macrophages. **a**. SCLC cells were collected 24 hours after irradiation (N = 1 experiment with 2 controls and 3 irradiated samples) and analyzed by bulk RNA sequencing (RNA-seq). Differentially expressed genes were obtained using DESeq2 using IHW for p value correction. Genes in red in the MA plot have a p-adjusted value < 0.05. **b**. GO of upregulated genes. **c**. GO of downregulated genes. See also Supplementary Tables 3, 4. **d**. Cytokine array with conditioned medium harvested from irradiated and control KP1 mouse SCLC cells. N = 1 experiment for each time point (the average of technical triplicates is shown). A different array was used for day 1 versus days 3 and 5. See also Supplementary Table 5. **e**. Representative immunofluorescence image of an in vitro phagocytosis assay with mouse bone marrow-derived macrophages (BMDMs) marked by F4/80 (red) and beads conjugated with FITC (green). The supernatant of irradiated KP1 cells was compared to non-irradiated cells. DAPI stains the DNA in blue. Scale bar, 100 μm. **f**. Quantification of (**e**). N = 1 experiment with triplicates. p = 0.0381. **g**. Flow cytometry analysis of an in vitro phagocytosis assay with BMDMs and KP3 mouse SCLC cells labeled with Calcein AM. N = 1 experiment with 6 technical replicates. **h**. Normalized migration ability of irradiated and control BMDMs (Mac.) cultured with conditioned medium of irradiated or control KP1 cells. N = 4 independent experiments with 2–3 replicates per experiment. *p = 0.0286. **i**. Normalized migration ability of BMDMs cultured with conditioned medium of irradiated (two doses) and control KP1 cells. N = 4 independent experiments with triplicates. *p = 0.0286, *p = 0.0286. **j**. Mouse KP1 control, *Csf1* knockout (KO) (see Fig. 5d–g), or *Csf1*/*Ccl2* KO SCLC cells were engrafted into the right flank of recipient mice, with control KP1 cells on the left flank. Only right-side of tumors were irradiated. **k**. Relative mRNA level by RT–qPCR for *Csf1* and *Ccl2* in Control and *Csf1*/*Ccl2* KO cells (n = 3 technical replicates). **l**. Growth curves of KP1 allografts as in (**j**) at the irradiated and non-irradiated sites. N = 1 experiment with n = 7 (RT + Anti-CD47 (Con) and RT + Anti-CD47 (*Csf1* KO)), 8 (Anti-CD47), or 9 (Control, RT, and RT + Anti-CD47 (*Csf1*/*Ccl2* KO) mice. Irradiated tumors: ****p < 0.0001, **p = 0.0049, non-irradiated tumors: ***p = 0.0006, **p = 0.0013, *p = 0.0281, ****p < 0.0001. **m**. KP1 cells were engrafted into the left flank of recipient mice. Only right-side of flanks (no tumors) were irradiated. Growth curves of KP1 allografts at the non-irradiated site. N = 1 experiment with n = 6 (Control, Anti-CD47) or n = 7 (RT, RT + Anti-CD47) mice. Two-tailed unpaired t-tests were performed on technical replicates in (**f**) and (**g**) with primary BMDM cultures. Two-tailed t-tests following one-way ANOVA were performed in (**h**) (p = 0.0006) and (**i**) (p = 0.0038). Two-tailed t-tests following two-way ANOVA were performed in (**l**) ((irradiated tumors: p < 0.0001, non-irradiated tumors: p < 0.0001) and (**m**) (p = 0.3077). Error bars represent SEM. *p < 0.05, **p < 0.01, ***p < 0.001, ****p < 0.0001. Source data

When we evaluated the transcriptional responses of mouse SCLC cells to irradiation in culture by bulk RNA sequencing, we found that the Gene Ontology (GO) biological processes associated with the top genes induced 24 hours after radiation were enriched for inflammation and stress response (Fig. 5a, Extended Data Fig. 7a–c and Supplementary Tables 1–4). Accordingly, cytokine arrays identified increased secretion of cytokines known to recruit and activate macrophages, including CSF1, CCL2 and MCP3 (also known as CCL7)^34^ in the supernatant of irradiated mouse SCLC cells compared to controls (Extended Data Fig. 7d and Supplementary Table 5). These results were validated at the RNA level in the same cell line, a second mouse SCLC cell line (KP3) and a human SCLC cell line (NCI-H82) (Fig. 5a,b). Accordingly, we found that the supernatant of irradiated mouse SCLC cells enhances the phagocytic ability of primary mouse bone marrow-derived macrophages ex vivo (Fig. 5a,c and Extended Data Fig. 7e–g), as well as their migratory ability (Extended Data Fig. 7h–i). These experiments in culture suggested that irradiation of SCLC cells may lead to the activation of antitumor macrophages, in part through secretion of stimulatory cytokines.

To test this model in vivo, we focused on CSF1 as a lead candidate based on our in vitro data with cytokine arrays and because CSF1 secretion by irradiated cancer cells has been shown to recruit macrophages to the irradiated tumor site in other contexts^35^. We found that knockout of *Csf1* alone (Fig. 5d–f) or *Csf1* and *Ccl2* (Extended Data Fig. 7j–l) specifically in SCLC cells at the irradiated site significantly decreased abscopal responses of wild-type SCLC cells on the contralateral side. This decrease in abscopal responses when irradiated cells were *Csf1* knockout was correlated with a reduction in the number of macrophages recruited to non-irradiated sites in the combination treatment group (Fig. 5g). In contrast, irradiation of healthy tissue was not sufficient to induce abscopal responses (Extended Data Fig. 7m). These data suggest that secretion of CSF1 and other inflammatory cytokines by SCLC cells at the irradiated tumor site contributes to the ability of macrophages to migrate to the non-irradiated site to induce abscopal responses.

### CD47 blockade is required at the non-irradiated site

**Fig. 6:**
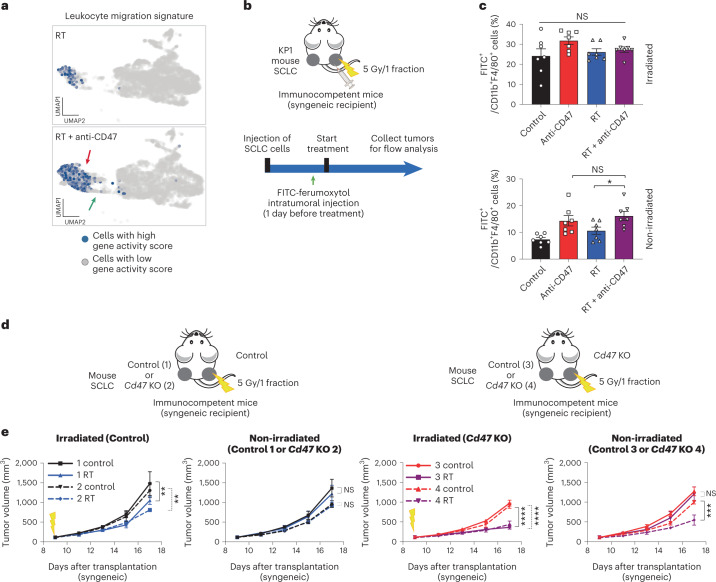
The abscopal effect is mediated by macrophages activated by CD47 blockade at the non-irradiated site. **a**, Representation of a gene signature associated with leukocyte migration in the scRNA-seq dataset. **b**, Schematic of in vivo macrophage migration assays using FITC-ferumoxytol, an iron oxide nanoparticle compound that is preferentially phagocytosed by tumor-associated macrophages. **c**, FITC-ferumoxytol was injected to right-side KP1 tumors as in **b** 24 h before the start of the treatment (RT or RT + anti-CD47). Shown is the quantification of CD11b^+^F4/80^+^ macrophages that are also FITC^+^ 5 d after the start of treatment. *n* = 1 experiment with *n* = 7 mice. *P = 0.0249. **d**, Mouse KP1 control and *Cd47* knockout SCLC cells were engrafted in the indicated combinations into both flanks of B6129SF1 immunocompetent syngeneic mice and only right-side tumors were irradiated; 1–2, wild-type tumors were irradiated; and 3–4, *Cd47* knockout tumors were irradiated. **e**, Growth curves of KP1 SCLC allografts as in **d** with the indicated treatments. *n* = 1 experiment with *n* = 5 (two control, two RT and three RT), *n* = 6 (one RT, three control, four control and four RT) or *n* = 7 (one control) mice. Irradiated (control), **P = 0.0083, **P = 0.0030; irradiated (*Cd47* KO), ****P < 0.0001; non-irradiated tumors, ***P = 0.0001. Two-tailed Student’s *t*-tests following one-way ANOVA were performed in **c** (irradiated tumors, *P* = 0.1332; non-irradiated tumors, *P* = 0.0017). Error bars represent s.e.m. *P < 0.05. Two-tailed Student’s *t*-tests following two-way ANOVA were performed in **e** (irradiated tumors, *P* < 0.0001; non-irradiated tumors, *P* < 0.0001; irradiated tumors, *P* < 0.0001; non-irradiated tumors, *P* < 0.000). Error bars represent s.e.m. *P < 0.05, **P < 0.01, ***P < 0.001, ****P < 0.0001. Source data

**Extended Data Fig. 8:**
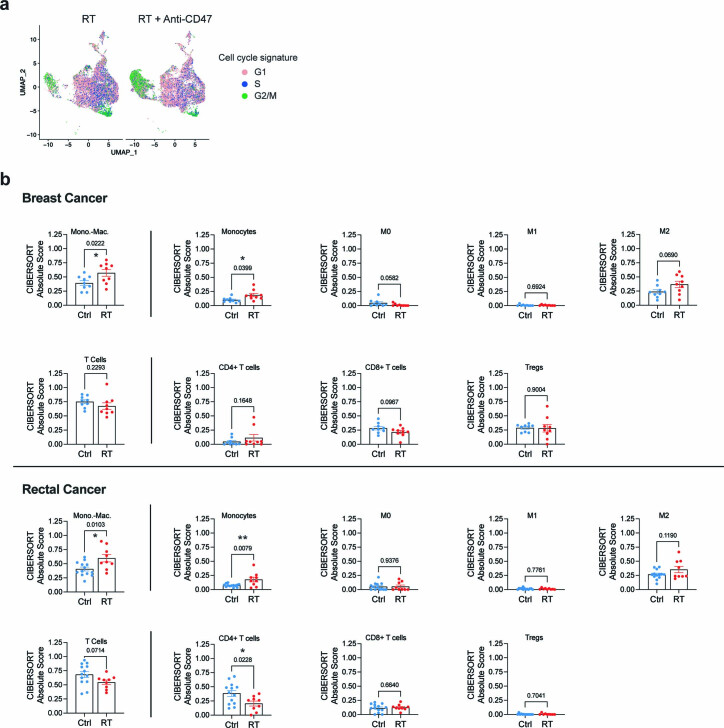
Increased numbers of monocytes/macrophages in response to irradiation in breast and rectal cancer. **a**. Signature for the cell cycle status of CD45 + hematopoietic cells in the scRNA-seq datasets as in Fig. 4d. **b**. Absolute abundance of monocytes-macrophages, monocytes, M0, M1, and M2 macrophages, T cells, CD4+, CD8+, and regulatory T cells based on CIBERSORTx enumeration in human breast cancer and rectal cancer. n = 9 (Ctrl, RT) in breast cancer. n = 13 (Ctrl) and n = 9 (RT) in rectal cancer. Two-tailed parametric paired (breast cancer study) or unpaired (rectal cancer study) t-tests were performed in **b** and the actual values are indicated in addition to the asterisks. *p<0.05, **p<0.01. Error bars represent SEM. Source data

**Extended Data Fig. 9:**
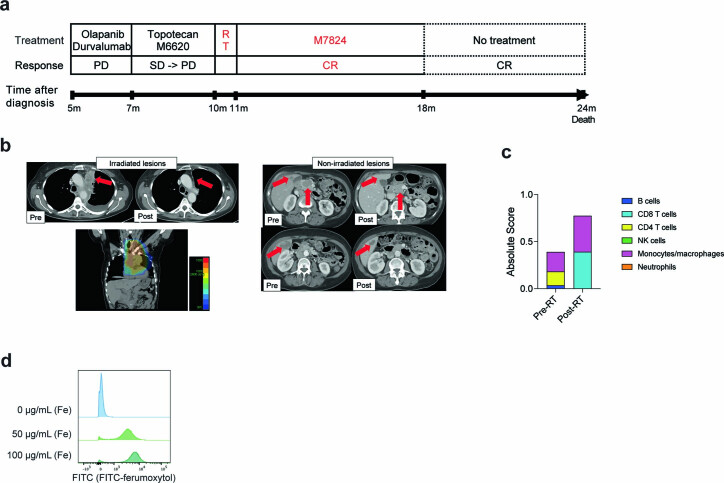
Possible abscopal response in a patient with SCLC associated with an increase in monocytes/macrophages, as well as CD8+ T cells. **a**. Timeline showing treatment and responses of mediastinal lymph nodes to treatment. The patient was a 59-year old female diagnosed with SCLC. She was initially treated with chemotherapy followed by olaparib and durvalumab (NCT02484404) and topotecan and berzosertib (NCT02487095). To relieve the symptoms caused by mediastinal lymph nodes, the patient was treated with radiotherapy (3000 cGy/10 fractions) and then treated with M7824 (for 7 months), a bifunctional fusion protein targeting PD-L1 and TGF-beta signaling. This patient died 12 months after radiotherapy due to aspiration pneumonia. **b**. CT images of pre- and 3 months post- radiotherapy and CT simulation image for radiotherapy planning. Although M7824 targets not only PD-1/PD-L1 but also TGF-beta signaling and is reported potent antitumor effects in some cancer types, M7824 did not show improved antitumor efficacy compared with historical results of immune checkpoint inhibitors in SCLC (NCT03554473). **c**. Absolute abundance of B cells, CD8+ T cells, CD4+ T cells, NK cells, monocytes/macrophages, and neutrophils based on CIBERSORT enumeration in cervical lymph node metastasis of pre- and post-radiotherapy. **d**. Representative flow cytometry analysis of J774A.1 mouse macrophage cells in culture 24 hours after adding FITC-ferumoxytol (Fe) to the culture medium at different concentrations. Experiment shown as a positive control of FITC-ferumoxytol phagocytosis by macrophages. Source data

We found no differences in the cell cycle signature of non-irradiated tumors between the two treatment groups in the scRNA-seq analysis, suggesting that the increase in macrophage numbers observed in non-irradiated tumors upon irradiation and CD47 blockade is not due to increased proliferation (Extended Data Fig. 8a). In contrast, the inflammatory macrophages/granulocytes whose numbers increased in the combination treatment group have a strong migratory signature (Fig. 6a), supporting the increased migratory potential of macrophages observed ex vivo (Extended Data Fig. 7h,i) and in tumors (Figs. 1c and 2h and Extended Data Figs. 1f,j, 4c,e and 6c). Analysis of clinical data collected from patients with breast and rectal cancer treated with radiotherapy suggested that irradiation stimulates the recruitment of macrophages to the tumor microenvironment (Extended Data Fig. 8b). While clinical trials of radiotherapy and CD47 blockade have not yet been pursued, we observed potential abscopal responses in a patient with SCLC treated with radiotherapy and M7824, a bifunctional fusion protein targeting the PD-L1 and transforming growth factor (TGF)-β pathway^36,37^ (Extended Data Fig. 9a,b). In this patient, we found an increase in monocytes/macrophages, as well as CD8^+^ T cells in post-radiotherapy tumors (Extended Data Fig. 9c). These data further support the possibility that infiltration of macrophages can play a role in abscopal responses in patients with SCLC.

To further evaluate the migratory potential of macrophages in vivo in response to CD47 blockade and irradiation, we labeled macrophages within the irradiated tumor through local injection of FITC-ferumoxytol, a fluorescently-tagged iron oxide nanoparticle that is preferentially phagocytosed by macrophages^38^ (Fig. 6b and Extended Data Fig. 9d). In these experiments, CD47 blockade alone and in the context of combination therapy led to more labeled macrophages at the non-irradiated sites compared to radiotherapy alone (Fig. 6c), indicating that CD47 blockade can potentiate not only phagocytosis but also migratory phenotypes in vivo.

These results raised the question of whether systemic blockade of CD47 was necessary for the observed abscopal effects. To address this question, we examined tumor growth in pairs of wild-type and *Cd47* knockout allografts, without systemic treatment with CD47-blocking antibodies. When irradiated tumors were composed of wild-type cells, no abscopal effects were observed whether the non-irradiated tumors were wild-type or *Cd47* knockout (Fig. 6d,e, conditions 1 and 2). When irradiated tumors were composed of *Cd47* knockout cells, abscopal effects were observed only when the non-irradiated tumors were also composed of *Cd47* knockout cells (Fig. 6d,e, conditions 3 and 4). These results indicate that systemic CD47 blockade is not necessary for abscopal effects but that it is required at both the irradiated and non-irradiated tumors.

### Abscopal responses upon CD47 blockade are not limited to SCLC

**Fig. 7:**
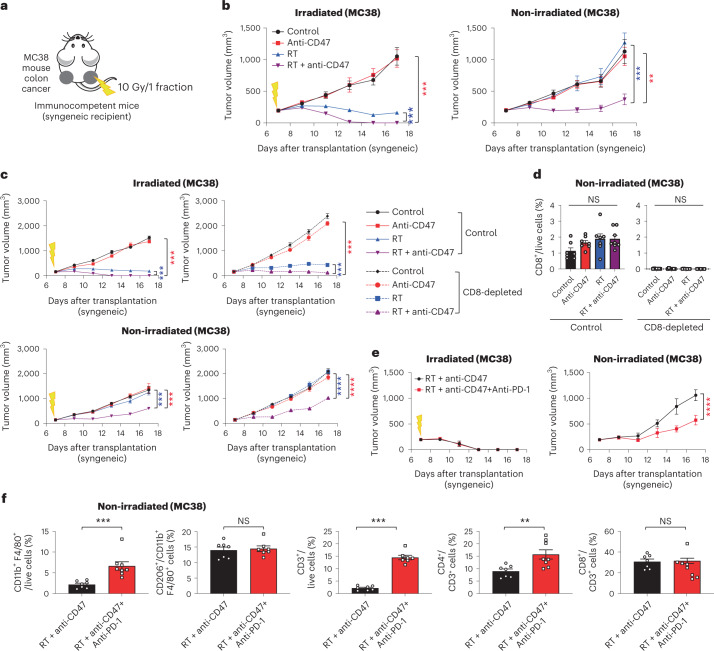
Activation of T cells by PD-1 blockade enhances abscopal effects upon radiation therapy and CD47 blockade in a colon cancer model. **a**, Mouse colon cancer MC38 cells were engrafted into both flanks of C57Bl/6 immunocompetent syngeneic mice and only right-sided tumors were irradiated. **b**, Growth curves of MC38 allografts with the indicated treatments in irradiated and non-irradiated tumors. *n* = 1 experiment with *n* = 6 (control, anti-CD47 and RT) or *n* = 7 (RT + anti-CD47) mice. Irradiated tumors, ***P = 0.0006, ***P = 0.0006; non-irradiated tumors, **P = 0.0015, ***P = 0.0002. **c**, As in **a** with MC38 allografts in C57BL/6 mice except that CD8^+^ T cells were depleted with anti-CD8 antibody treatment. *n* = 1 experiment with *n* = 7 mice except RT + anti-CD47 (*n* = 8 mice). Irradiated tumors (control), ***P = 0.0002, ***P = 0.0002; irradiated tumors (CD8-depleted), ***P = 0.0006, ***P = 0.0006; non-irradiated tumors (control), ***P = 0.0003, ***P = 0.0003; non-irradiated tumors (CD8-depleted), ****P < 0.0001. **d**, Quantification of tumor-infiltrating CD8^+^ T cells in non-irradiated tumors from **f**. *n* = 1 experiment with *n* = 7 mice except RT + anti-CD47 (*n* = 8 mice). **e**, Growth curves of MC38 allografts with the indicated treatments in irradiated and non-irradiated tumors. *n* = 1 experiment with *n* = 7 (RT + anti-CD47) or *n* = 8 (RT + anti-CD47 + anti-PD-1) mice (two tumors per mouse). ****P < 0.0001. **f**, Quantification of tumor-infiltrating macrophages, M2-like macrophages, total T cells, CD4^+^ T cells and CD8^+^ T cells in non-irradiated tumors from **e** by flow cytometry. *n* = 1 experiment with *n* = 7 (RT + anti-CD47) or 8 (RT + anti-CD47 + anti-PD-1) mice. ***P = 0.0003, ***P = 0.0006, **P = 0.0078. Two-tailed Student’s *t*-tests following two-way ANOVA were performed in **b** (irradiated tumors, *P* < 0.0001; non-irradiated tumors, *P* < 0.0001), **c** (irradiated tumors, *P* < 0.0001; non-irradiated tumors, *P* < 0.0001) and **e** (irradiated tumors, *P* = 0.7841; non-irradiated tumors, *P* < 0.0001). Two-tailed Student’s *t*-tests following one-way ANOVA were performed in **d** (control, *P* = 0.0908; CD8-depleted, *P* = 0.4161). Two-tailed Student’s *t*-tests were performed for **f**. Error bars represent s.e.m. *P < 0.05, **P < 0.01, ***P < 0.001, ****P < 0.0001. Source data

**Extended Data Fig. 10:**
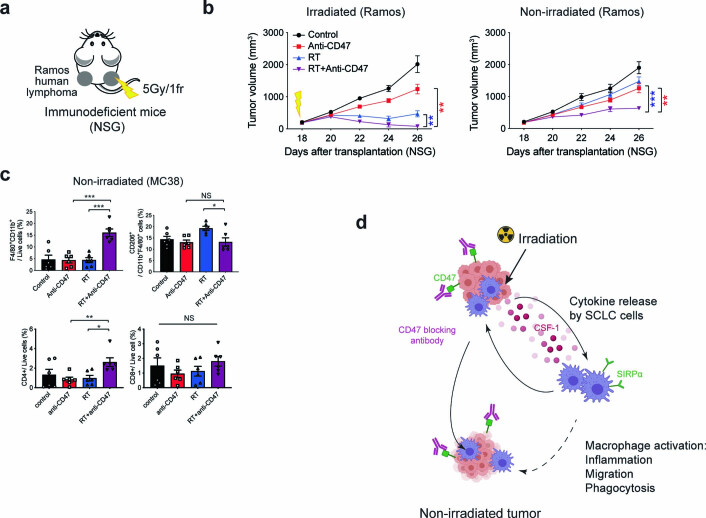
T-cell independent abscopal effects upon radiation therapy and CD47 blockade in cancer models. **a**. Human Ramos lymphoma cells were engrafted into both flanks of NSG immunodeficient mice and only right-side tumors were irradiated. **b**. Growth curves of Ramos xenografts. N = 1 experiment with n = 6 mice. Irradiated tumors: **p = 0.0022, **p = 0.0047, non-irradiated tumors: **p = 0.0021, ***p = 0.0002. **c**. MC38 allografts in C57Bl/6 mice were treated with the indicated treatments (as in Fig. 7a,b) and tumor-infiltrating macrophages (F4/80 + CD11b+), CD206 + M2-like macrophages, CD4+ T cells and CD8 + T cells were quantified by immunostaining. N = 1 experiment with n = 6 tumors. CD11b + F4/80+: ***p = 0.0001, ***p = 0.0001, CD206+: *p = 0.0158, CD4+: **p = 0.057, *p = 0.0110. **d**. Proposed model for macrophage-mediated abscopal effects. Irradiation of tumors results in the secretion of inflammatory molecules such as CSF1 and other cytokines that can activate macrophages. Blockade of CD47 further activate the migratory and phagocytic activity of macrophages. CD47 blockade is critical at both the irradiated and non-irradiated tumor sites but not required systemically. Activated macrophages can migrate from the irradiated site to the non-irradiated site, and possibly also directly from the circulation. Not shown in the model, T cells can cooperate with macrophages to enhance antitumor responses. Two-tailed t-tests following two-way ANOVA were performed in (**b**) (irradiated tumors: p < 0.0001, non-irradiated tumors: p < 0.0001). Two-tailed t-tests following one-way ANOVA were performed in (**c**) (CD11b + F4/80+: p < 0.0001, CD206+: p = 0.0121, CD4+: p = 0.0217, CD8+: p = 0.4010) Error bars represent SEM. *p < 0.05, **p < 0.01, ***p < 0.001, ****p < 0.0001. Source data

These observations in mouse and human preclinical models of SCLC and the increased presence of monocytes/macrophages in breast and colon tumors after irradiation raised the question whether abscopal responses could also be induced in other cancer models in response to the combination of radiotherapy and CD47 blockade. Indeed, T-cell independent abscopal effects upon radiotherapy and CD47 blockade were also observed in a xenograft model of lymphoma (Ramos cells, grown in immunodeficient NSG mice) (Extended Data Fig. 10a,b). Similar results were observed in an allograft model of colon cancer (MC38, grown in syngeneic C57BL/6J mice), along with the recruitment of macrophages to the non-irradiated site (Fig. 7a–d and Extended Data Fig. 10c), indicating that the induction of abscopal responses observations is not limited to SCLC. In contrast to SCLC tumors, which rarely respond strongly to T-cell checkpoint inhibitors, MC38 cells are a well-established model to investigate the responses of cytotoxic T cells against cancer cells, including PD-1/PD-L1 blockade^39^. PD-1 blockade enhanced the long-term abscopal antitumor effects of irradiation and CD47 blockade in this model (Fig. 7e), which was accompanied by an increase in both macrophages and T cells (Fig. 7f). Thus, while T cells are not required for abscopal responses upon CD47 blockade, the triple therapy (radiotherapy, CD47 blockade and PD-1 blockade) may have additional antitumor effects in some contexts.

## Discussion

Radiation-induced systemic antitumor effects were first defined by Mole in 1953 (ref. ^40^) and have been associated with activation of systemic immunity^24^. Emerging evidence suggests that immune-checkpoint inhibitors such as PD-1/PD-L1 inhibitors and CTLA-4 blockade can facilitate systemic effects by activating the adaptive immune system, including cytotoxic CD8^+^ T cells^23–25,41^; however, thus far, abscopal responses in the clinic remain relatively rare^8,42^ and despite continuing preclinical investigation, it has proven difficult to reproducibly achieve abscopal responses in the clinic. The radiation dose and fractionation applied along with additional chemotherapy as well as the host immune microenvironment each seem to influence induction of a subsequent abscopal response^43^. Our studies to systematically deconstruct the cellular mechanism underlying abscopal responses using in vivo models of SCLC and other cancer types indicate that these responses can be achieved potently and reproducibly by blocking CD47 and activating macrophages (Extended Data Fig. 10d).

While T-cell based immunotherapies have led to improved overall survival rates in patients with SCLC in combination with standard-of-care treatment^15,16^, the long-term beneficial effects of these strategies remain limited to a small number of patients. Low expression of major histocompatibility complex I molecules on the surface of SCLC cells and the subsequent lack of tumor antigen presentation^44^ as well as a generally immunosuppressive tumor microenvironment with few infiltrating cytotoxic CD8^+^ T cells^45^ may explain the limited efficacy of treatment with anti-PD-1/PD-L1 (ref. ^46^). In contrast, SCLC tumors can have high levels of macrophage infiltration^20,47^. While tumor-associated macrophages often have protumor effects in part via immune suppressive mechanisms^48,49^, greater numbers of macrophages in SCLC tumors have been associated with improved survival^50^. Given that radiotherapy is a common treatment option for patients with SCLC and that several clinical trials to test anti-CD47 antibody efficacy against hematologic malignancies and solid tumors are ongoing^51–54^, our preclinical findings could be readily tested in the clinic in patients with SCLC and other cancers. In patients with SCLC, the combination of radiotherapy and CD47 blockade may improve treatment of primary tumors or metastases treated directly with radiotherapy but could also reduce the growth of distant lesions in patients with widespread metastatic disease that are difficult to irradiate in total^14^.

In tumors, CD47 blockade may increase phagocytosis in M1-like but also M2-like macrophages^55^, thus possibly promoting an antitumor microenvironment. Notably, our observations also suggest that it may be possible to alleviate some of the adverse side effects of systemic CD47 blockade such as anemia^52^ by targeting the delivery of CD47-blocking agents to tumors. Our data further suggest that it may also be possible in future studies to identify combinations of cytokines that mimic in part the effects of radiation therapy and/or CD47 blockade to potentiate the effects of these treatments and/or limit their side effects. Recently, other ‘don’t-eat-me’ cell surface molecules such as CD24, PD-L1 and β2-microglobulin, have been reported^56–58^. It remains to be determined whether blockade of these molecules also activate abscopal responses in SCLC and other tumors via mechanisms similar to CD47 blockade.

Loss of the CD47 ligand SIRPα in macrophages can lead to the activation of antigen-specific cytotoxic T cells following radiotherapy in preclinical models^59^. The combination of radiotherapy and inhibition of PD-L1 can lead to activation of T cells and but also phagocytic macrophages in glioblastoma preclinical models^60^. Thus, while we have demonstrated the potential of T-cell-independent abscopal effects through combination of radiotherapy and CD47 blockade, there may nevertheless be further interactions between innate and adaptive immune responses that could be clinically important. Activating both T cells and macrophages may further enhance systemic antitumor effects in the context of radiotherapy in patients. These findings along with the number of clinical trials currently evaluating the efficacy of radiotherapy and immunotherapies, such as anti-PD-1/PD-1, suggest that future studies should investigate whether abscopal effects can be recapitulated clinically using this strategy. Furthermore, additional work is needed to evaluate the generality of this response in other cancer models and to determine the relative role of the innate and acquired immune systems in these effects.

## Methods

### Ethics statement

Mice were maintained according to practices prescribed by the National Institutes of Health (NIH) at Stanford’s Research Animal Facility and by the Institutional Animal Care and Use Committee at Stanford. Additional accreditation of Stanford animal research facility was provided by the Association for Assessment and Accreditation of Laboratory Animal Care. The patient with SCLC was provided written informed consent. The trial was conducted under an institutional review board approval (National Cancer Institute (NCI) identifier, 18-c-0110).

### Cell lines and culture

Human NCI-H82, NCI-H69 and NCI-H526 SCLC cells were obtained from the American Type Culture Collection. NJH29 human SCLC cells were described before and were propagated in our laboratory^61^. RNA-seq analysis of NJH29 was performed in our laboratory^62^. NCI-H82 and NJH29 cells belong to the SCLC-N subtype, NCI-H69 cells the SCLC-A subtype and NCI-H526 cells to the SCLC-P subtype^63^. *Rb/p53* mutant mouse SCLC KP1 and KP3 cells were previously described and propagated in our laboratory^64^ (SCLC-A subtype). All SCLC cells and Ramos cells were cultured in RPMI-1640 supplemented with 10% fetal bovine serum (Hyclone), 1× GlutaMax (Invitrogen) and 100 U ml^−1^ penicillin and 100 µg ml^−1^ streptomycin (Invitrogen). Cell lines were grown in suspension and dissociated by gently pipetting. MC38 cells and J774 cells were cultured in DMEM supplemented with 10% fetal bovine serum (Hyclone) and 100 U ml^−1^ penicillin and 100 µg ml^−1^ streptomycin (Invitrogen). Cell lines were cultured in humidified incubators at 37 °C with 5% carbon dioxide. All cell lines are routinely tested (Lonza) and confirmed to be free of mycoplasma contamination.

### Knockout cells

KP1 *Cd47* KO and control cells were previously described^20^. The single guide RNAs (sgRNAs) for *CD47*, *Csf1* and *Ccl2* were purchased from Synthego. We added 12 µl of SE buffer (Lonza, V4XC-1032) to each well of a 96-well v-bottom plate. Then 3 µl of sgRNA (300 pmol) was added to the SE buffer. An aliquot of 0.5 µl of Alt-R S.p. Cas9 (Integrated DNA Technologies, 1081059) was added to 10 µl of SE buffer. Next, Cas9 was added to the sgRNA solution, mixed thoroughly and incubated at 37 °C for 15 min to form the ribonucleoproteins. NJH29 cells or KP1 cells were pelleted, counted and resuspended to 10^6^ cells per reaction in 5 µl of SE buffer. Cells and the ribonucleoprotein solution were added to each nucleofection well chamber. Cells were immediately nucleofected using the Lonza 4D-NucleofectorTM X Unit (Lonza, AAF-1002X) with the EN150 program. After nucleofection, warm RPMI medium was added to the cells. The cells were incubated at 37 °C for 15 min and then transferred to a 24-well plate with 1 ml RPMI medium. Editing efficiency was evaluated 4 d later by FACS or RT–qPCR.

### In vivo SCLC models

All experiments using mice were performed as per protocols by the NIH at Stanford’s Research animal facility and by the Institutional Animal Care and Use Committee at Stanford. Nod.Cg-Prkdc^scid^IL2rg^tm1WjI^/SzJ (NSG) mice (Jackson Laboratories, stock no. 005557) were used for experiments in immunodeficient recipients. B6.129S F1 mice (Jackson Laboratories, stock no. 101043) were used for experiments in immunocompetent recipients. Mice were engrafted with 10^6^ cancer cells in antibiotic-free serum-free medium with a 1:1 mixture of Matrigel (BD Matrigel, 356237) at 6–15 weeks of age. Both male and female were used (no selection for sex of mice). The tumors did not exceed the 1.75-cm diameter permitted by our animal protocol.

For allograft models, tumors were allowed to grow for 10–14 d and then mice were randomized into treatment groups with PBS or 200 µg anti-mouse CD47 antibody (MIAP410, Bio X Cell) every other day and/or radiation. The radiation dose was 5 Gy unless otherwise stated. For KP1 *Cd47* KO allografts, tumors were irradiated when the average tumor size reached around 150–300 mm^3^, day 10 for KP1 control and day 14 for KP1 *Cd47* KO cells in NSG mice and day 11 for KP1 control and day 13 for KP1 *Cd47* KO cells in B6.129 S F1 mice. In the experiments with subcutaneous tumors and liver tumors in the same mice, 10^6^ KP1 cells in PBS were intravenously injected and 10^6^ KP1 cells in antibiotic-free serum-free medium with a 1:1 mixture of Matrigel were engrafted into the right flanks of B6.129S F1 mice on the same day. Tumors were allowed to grow for 8 d and then mice were randomized into treatment groups with PBS or 200 µg anti-mouse CD47 antibody every other day and/or radiation to liver metastases. In the experiment with normal tissue irradiation, 10^6^ KP1 cells in antibiotic-free serum-free medium with a 1:1 mixture of Matrigel were engrafted into the left flanks of B6.129S F1 mice. Tumors were allowed to grow for 6 d and then mice were randomized into treatment groups with PBS or 200 µg anti-mouse CD47 antibody every other day and/or radiation (5 Gy/1 fraction) to right flanks (without tumors).

For xenograft models, tumors were allowed to grow for 10–14 d and then the mice were randomized into treatment groups with PBS or 400 µg anti-human CD47 antibody (B6H12, Bio X Cell) every day and/or radiation. The radiation dose was 5 Gy unless otherwise stated. Mice were treated with 10 mg kg^−1^ anti-CD8a antibody (2.43, Bio X Cell) twice per week for CD8^+^ T-cell depletion, 10 mg kg^−1^ anti-CSF1 antibody (5A1, Bio X Cell) three times per week for macrophage depletion^35^ and 10 mg kg^−1^ anti-PD-1 antibody (RMP1-14, Bio X cell) twice per week for T-cell experiments.

For all treatment models, therapeutic agents were administered by intraperitoneal injection. For all models, tumor growth was monitored by tumor dimension measurements that were used to calculate tumor volume. Tumor volumes were calculated as 0.5 × length × width^2^.

### In vivo colon cancer model

C57BL/6J mice (Jackson Laboratories, stock no. 000664) were used for experiments in immunocompetent recipients. Mice were engrafted with 0.5 × 10^6^ MC38 cells in antibiotic-free serum-free medium with 1:1 mixture of Matrigel at 6–15 weeks of age. Tumors were allowed to grow for 7 d and then mice were randomized into treatment groups with PBS or 200 µg anti-mouse CD47 antibody every other day and/or radiation (10 Gy/1 fraction).

### In vivo lymphoma model

NSG mice were used for experiments in immunodeficient recipients. Mice were engrafted with 2 × 10^6^ Ramos cells in antibiotic-free serum-free medium with 1:1 mixture of Matrigel at 6–15 weeks of age. Tumors were allowed to grow for 18 d and then mice were randomized into treatment groups with PBS or 400 µg anti-human CD47 antibody (B6H12, Bio X Cell) every day and/or radiation (5 Gy/1 fraction).

### Radiation

Animal irradiation was performed using a PXi X-rad SmART cabinet irradiator (Precision X-Ray)^65^. Mice were anesthetized using isoflurane through a nose cone supplied to the animal stage. Computed tomography images were acquired using a beam energy of 40 kVp, a beam filter of 2 mm Al and a voxel size of 0.2 mm. Treatment planning was performed with the RT image software package, v.3.13.1. A 10-mm collimator was used to target tumors while sparing adjacent normal tissue. In the experiment with normal tissue irradiation, the same 10-mm collimator was used, irradiating the flank with no tumor. Therapeutic irradiations were performed using an X-ray energy of 225 kVp and a current of 13 mA producing a dose rate of 241 cGy min^−1^ at the isocenter. The procedure described by AAPM TG-61 was used to commission and calibrate the irradiator and to ensure dosimetric accuracy through biannual quality assurance, using ion chamber and radiochromic film measurements.

### RNA sequencing and analysis

For RNA-seq analysis, cell pellets were collected and sent to Novogene for RNA extraction and Illumina sequencing. Reads were quantified based on the mouse reference genome mm10 using Salmon^66^ using default settings. Differentially expressed genes were obtained using DESeq2 (ref. ^67^) using Independent Hypothesis Weighting (IHW) for *P* value correction^68^. Plots were generated ggplot2 (https://ggplot2.tidyverse.org). Genes were selected by filtering for log_2_ fold change (FC) >1.5 or <−1.5 with corrected *P* value <0.05. GO pathway analysis was performed using Metascape^69^.

### Single-cell RNA sequencing and analysis

NSG mice were engrafted with 10^6^ mouse SCLC KP1 cells in antibiotic-free serum-free medium with a 1:1 mixture of Matrigel in both flanks. Tumors were allowed to grow for 7 d and then mice were randomized into treatment groups with PBS or 200 µg anti-mouse CD47 antibody every other day and radiation (5 Gy/1 fraction). After 6 d, tumors were removed and single-cell suspensions were prepared. Cells were resuspended in PBS, counted, Fc receptors were blocked with CD16/32 antibody (BioLegend) and cells were stained with Brilliant Violet 421 anti-mouse CD45 antibody (1:200 dilution, 103133, 30-F11, BioLegend) and 4,6-diamidino-2-phenylindole (DAPI). CD45-positive, DAPI-negative cells were selected with flow cytometry. Then, 5,000–10,000 cells per samples were barcoded and libraries were generated using the V2 10x Chromium system. The samples were aggregated and sequenced using NovaSeq with a target of 30,000 reads per cell.

For pre-processing of scRNA-seq data, the scRNA-seq data from each sample were individually pre-processed using the CellRanger v.6.0 pipeline. Briefly, fastq files generated through the mkfastq function were aligned to the mm10 reference genome using the CellRanger count function with default parameters. Next, the CellRanger aggr function was utilized to normalize and combine the feature–barcode matrices of samples belonging to the same treatment group. The downstream analysis on these datasets was performed using the Seurat v.4 package.

For quality control, batch correction and sample integration, Seurat objects corresponding to each treatment condition were generated separately using the aggregated filtered barcode matrix files (CellRanger output) and initial quality control steps were performed to remove low-quality cells and doublets. Cells with >10% mitochondrial reads and <500 and >6,000 expressed genes were removed from the Seurat objects alongside heterotypic doublets identified using the DoubletFinder package. Next, the normalization and scaling of the count data was performed with the sctransform function. Additionally, during this normalization step, the effect of confounding factors such as differences in cell quality was regressed out and the top variable features were determined. Using the 3,000 variable genes thus identified as input, principal-component analysis was performed and the top 30 principal components were retained which explained 65% of the variance. Lastly, the two Seurat objects (RT and RT + CD47 blockade) were integrated based on ‘anchor’ cell populations found in both conditions, which yielded a matrix of 20,608 genes by 24,710 cells profiled from the two treatment conditions (RT, 12,261 cells; RT + CD47 blockade, 12,449 cells). The median number of molecules/cells was 11,898 and the median number of genes/cells was 3,124. Subsequent cell clustering (resolution of 0.5) and visualization of the cells in UMAP space were performed on the integrated object.

For establishing cluster identity and to unbiasedly determine the cell type of the 19 distinct clusters identified in the integrated dataset, the scMCA package was utilized to map the identity to each cell to the publicly available mouse cell atlas (MCA v.2.0.0). To speed up the analysis, the integrated Seurat object was randomly downsampled to 14,000 cells and scMCA was run on the top 3,000 highly variable genes. While each cell in the single-cell dataset was mapped to three possible cell identities in MCA, the highest confidence identity was considered for downstream analysis. Next, the most frequent cell type in each cluster was determined, which was then used to rename cluster identity. Macrophage clusters that had more than one predominant cell type (>30%) were labeled as ‘mixed macrophages’. This analysis also identified two small populations which corresponded to contaminating cancer cells and T cells in our dataset. These two clusters were removed before further downstream analysis. The cell-type identity thus determined was further confirmed by looking at marker genes’ expression pattern in our dataset. To identify differential genes, the differentially expressed gene in each cluster and those between the two treatment conditions, were determined using the FindMarkers() function implemented through Seurat. This analysis uses a nonparametric Wilcoxon test to determine marker genes, which based on our cutoff had avglog_2_(FC) > 0.5 and adjusted *P* value <1 × 10^−5^.

Identifying cells with active gene sets: The AUCell package was used to identify cell populations expressing specific gene sets. AUCell analysis was performed on the integrated gene expression matrix obtained after SCT normalization. The expression matrix was split according to treatment condition (RT versus RT + CD47) and analyzed individually. The two active gene sets assessed in this manuscript were ‘Leukocyte migration upon inflammation’ (obtained from msigDB) and the ‘Fibrogenic macrophage’ gene set obtained from Chan et al. (top 50 marker genes of this population was used in the analysis). Cell ranking, area under the curve calculation and cell assignment were implemented using default parameters. To obtain the final plots showing gene set projection on the UMAP, an area under the curve threshold of 0.25 was used for the leukocyte gene set and a threshold of 0.4 was used for the fibrogenic gene set (for both treatment conditions). The proliferation status of the cells in our dataset was determined using the CellCycleScoring() function of Seurat. Briefly, the function calculates a G2/M and S phase score to each cell based on the combined expression of a two pre-curated gene sets ‘g2m genes’ and ‘s genes’, respectively. Based on this scoring, the function assigns the G1, S or G2/M cell cycle phase to each cell (with the G1 phase corresponding to cells not expressing either S or G/2M genes). We used this cell-cycle phase assignment to generate the plots depicted in the figures.

### Determining immune cell abundance in patient tumor samples

CIBERSORTx^70^ (https://cibersortx.stanford.edu/) was used to enumerate immune cell abundance in patient tumor samples that underwent transcriptomic profiling. Processed data from GSE59733 (refs. ^71,72^) and GSE15781 (ref. ^73^) were downloaded from Gene Expression Omnibus (GEO)^74^. Genes were annotated and for genes with multiple probes, only the probe with the maximum mean value across all samples was included. Unannotated genes were excluded. Mixture files were analyzed with CIBERSORTx and the ‘Impute Cell Fractions’ Analysis Module was applied using the LM22 (ref. ^75^) signature matrix with absolute mode, batch correction, quantile normalization and 1,000 permutations. Values for merged cell subsets (monocytes/macrophages, T cells and CD4^+^ T cells) were determined by combining values of respective individual subsets.

### Macrophage differentiation and phagocytosis assays

Mouse macrophages were differentiated as previously described^20^. Briefly, mouse macrophages were differentiated from the bone marrow of B6.129S F1 mice. Unfractionated bone marrow cells were cultured in RPMI + GlutaMax with 10% fetal bovine serum, 100 U ml^−1^ penicillin and 100 µg ml^−1^ streptomycin and 10 ng ml^−1^ murine M-CSF (Peprotech). In vitro phagocytosis assays were performed as previously described^20^. Briefly, SCLC cell lines labeled with calcein AM (Invitrogen) or FITC-conjugated beads (Cayman, 500290) were used as targets. Macrophages were washed twice with PBS, then incubated with 1× TrypLE for approximately 10 min in a humidified incubator at 37 °C. Macrophages were removed from the plates using gentle pipetting, then washed twice with serum-free RPMI. Phagocytosis reactions were carried out using 50,000 macrophages and 100,000 target cancer cells for 2 h in a humidified 5% CO_2_ incubator at 37 °C in 96-well U-bottom plates. After co-culture, cells were washed with PBS and stained with BV785-labeled anti-CD11b (CloneM1/70, BioLegend) to identify mouse macrophages. Assays were analyzed by flow cytometry using a LSRFortessa (BD Biosciences). Phagocytosis was measured as the number of CD11b^+^ calcein AM^+^ macrophages, quantified as a percentage of total CD11b^+^ macrophages.

### Migration assay

In vitro migration ability was assessed in the Transwell migration system with membranes with 8-μm pore size (BioVision, K906). Mouse macrophages were cultured in the top wells and SCLC conditioned medium with or without irradiation was added in the bottom wells. Migrated cells were lysed and detected 48 h after using a BioTek plate reader with Gen5 software.

### Flow cytometry

To create cell suspensions, tumors were removed, finely chopped and suspended in PBS. Tumors were digested with collagenase/dispase for 30 min at 37 °C then filtered through a 40-µm mesh. Cells were resuspended in red blood cell lysis buffer for 1 min at room temperature. Cells were resuspended in PBS, counted, Fc receptors were blocked with CD16/32 antibody (BioLegend) and then 10^6^ cells were stained with conjugated antibody cocktail for 20 min on ice. Cells were washed two times in PBS and then resuspended for flow cytometry analysis.

### In vivo phagocytosis analysis

Mice were engrafted with 10^6^ NCI-H82-GFP-luc cancer cells in antibiotic-free serum-free medium with 1:1 mixture of Matrigel (BD Matrigel, 356237) at 6–15 weeks of age. Tumors were allowed to grow for 10–14 d and then the mice were randomized into treatment groups with PBS or 400 µg anti-human CD47 antibody (B6H12, Bio X Cell) every day and/or radiation. After 6 d, tumors were removed and single-cell suspensions were prepared as described above. Cells were resuspended in PBS and counted, Fc receptors were blocked with CD16/32 antibody (BioLegend) and then 10^6^ cells were stained with conjugated antibody cocktail for 20 min on ice. Cells were washed twice in PBS and then resuspended for flow cytometry analysis. Phagocytosis was measured as the percentage of CD11b^+^F4/80^+^ macrophages that were also GFP^+^.

### In vivo migration analysis

Mice were engrafted with 10^6^ cancer cells in antibiotic-free serum-free medium with 1:1 mixture of Matrigel (BD Matrigel, 356237) at 6–15 weeks of age. Tumors were allowed to grow for 10–14 d and then the mice were randomized into treatment groups with PBS or 200 µg anti-mouse CD47 antibody (MIAP410, Bio X Cell) every other day and/or radiation. FITC-ferumoxytol was injected to the right side of tumors 24 h before the start of radiation. After 6 d, tumors were removed and single-cell suspensions were prepared as described above. Cells were resuspended in PBS and counted, Fc receptors were blocked with CD16/32 antibody (BioLegend) and then 10^6^ cells were stained with conjugated antibody cocktail for 20 min on ice. Cells were washed twice in PBS and then resuspended for flow cytometry analysis. In vivo migration was measured as the percentage of CD11b^+^F4/80^+^ macrophages that were also FITC^+^ in the non-irradiated tumors.

### Immunoblot

Cells were lysed and sonicated in RIPA buffer (Pierce) with protease and phosphatase inhibitor tablets (Roche). Protein concentration was measured using the Pierce BCA Protein Assay kit (Thermo Fisher Scientific) and 30 μg of protein was analyzed by immunoblot.

### Cytokine profiling

Mouse cytokine secretion was assessed in vitro. Cells were irradiated with 5 Gy and cultured for 24 h, 72 h or 120 h and then supernatants were collected and stored at −80 °C. Mouse cytokines were analyzed by the Stanford University Human Immune Monitoring Center using a Luminex 38-plex mouse cytokine array.

### Immunostaining

Tumor samples were fixed in 10% neutral buffered formalin and embedded in paraffin before staining with H&E or immunostaining. Tumor sections were dewaxed, antigen retrieval was performed with proteinase K treatment (20 µg ml^−1^ for 15 min; Thermo Fisher Scientific, 25530049) and sections were stained with rat anti-mouse F4/80 antibody (1:50 dilution, BM8, Invitrogen). DAB was developed until precipitation was noted in specific areas of tumor sections using the HRP/DAB kit (Abcam, av64238). To block non-specific signal and increase sensitivity, the Avidin/Biotin Blocking kit (Vector Laboratories) and TSA Biotin kits (PerkinElmer) were used.

### RT–qPCR

Total RNA was extracted using the RNAeasy Mini kit (QIAGEN). For RT–qPCR, 1 µg of total RNA was used to make cDNA using the NEB ProtoScript cDNA synthesis kit and cDNA was diluted 1:20 before use. Primers used are listed in Supplementary Table 6.

### Potential abscopal responses in a patient with small cell lung cancer

The patient shown in Extended Data Fig. 9a–c was initially treated with four cycles of carboplatin and etoposide. The patient had a recurrence in a left breast mass, mediastinal lymphadenopathy, liver lesions and a pancreatic mass, 76 d after completion of platinum-based chemotherapy. Subsequently the patient was enrolled in two investigational combination treatment clinical trials (durvalumab and olaparib; ClinicalTrial.gov identifier, NCT02484404 and berzosertib and topotecan, NCT02487095). After palliative radiation to growing mediastinal lymph node lesions (3,000 cGy/10 fractions), the patient was treated on a clinical trial of M7824 (bintrafusp alfa), a first-in-class bifunctional fusion protein composed of the extracellular domain of the TGF-βRII receptor (a TGF-β ‘trap’) fused to a human IgG1 monoclonal antibody blocking PD-L1 administrated at a dose of 1,800 mg m^−2^ intravenously every 3 weeks until isolated disease progression in the brain 7 months later. The patient provided written informed consent. The trial was conducted under an institutional review board approval (NCI identifier, 18-c-0110). Tumor samples of metastatic left cervical lymphadenopathy were collected before and after radiation by experienced interventional radiologists at the NIH for research purposes. Tumor RNA was sequenced and normalized to log_2_-transformed Trimmed mean of M values normalized fragments per kilobase of exon per million reads, mapped as previously described^76^. To deconvolute bulk gene expression data to immune subsets and SCLC transcriptomic subtype^77^, we applied CIBERSORTx with default parameters^70^. For immune subset analysis, we used the LMP6 gene set and weight^78^.

### Statistical and reproducibility

Data collection and analysis were not performed blind to the conditions of the experiment. Statistical significance was assayed with GraphPad Prism software. Data are represented as mean ± s.e.m. *P < 0.05, **P < 0.01, ***P < 0.001, ****P < 0.0001, NS not significant. Tests used are indicated in figure legends. No statistical method was used to predetermine sample sizes, but our sample sizes are similar to those reported in previous publications^20,56^. Data distribution was assumed to be normal, but this was not formally tested. No data were excluded from the analysis. To compare growth curves, we used two-way ANOVA followed by Student’s *t*-tests. When comparing more than two groups, we first performed one-way ANOVA, followed by Student’s *t*-tests. If the *F*-test for variance showed a significantly different distribution between two groups being compared (*F*-test *P* < 0.05), the nonparametric Mann–Whitney *P* value was reported instead of the Student’s *t*-test *P* value.

### Reporting summary

Further information on research design is available in the Nature Portfoilio Reporting Summary linked to this article.

### Supplementary information

Reporting Summary.

Supplementary Table Supplementary Tables 1–6.

## Data Availability

RNA-seq and scRNA-seq data are available on GEO under accession code GSE156106. Source data are provided with this paper. All other data supporting the findings of this study are available from the corresponding authors upon request.

## Source data

Source Data Fig. 1 Statistical Source Data.
Source Data Fig. 2 Statistical Source Data.
Source Data Fig. 3 Statistical Source Data.
Source Data Fig. 4 Statistical Source Data.
Source Data Fig. 5 Statistical Source Data.
Source Data Fig. 6 Statistical Source Data.
Source Data Fig. 7 Statistical Source Data.
Source Data Extended Data Fig. 1 Statistical Source Data.
Source Data Extended Data Fig. 2 Statistical Source Data.
Source Data Extended Data Fig. 3 Statistical Source Data.
Source Data Extended Data Fig. 3 Unprocessed immunoblots.
Source Data Extended Data Fig. 4 Statistical Source Data.
Source Data Extended Data Fig. 5 Statistical Source Data.
Source Data Extended Data Fig. 6 Statistical Source Data.
Source Data Extended Data Fig. 7 Statistical Source Data.
Source Data Extended Data Fig. 8 Statistical Source Data.
Source Data Extended Data Fig. 9 Statistical Source Data.
Source Data Extended Data Fig. 10 Statistical Source Data.
