# Supplementary material for: Radiotherapy in combination with CD47 blockade elicits a macrophage-mediated abscopal effect
Source: Nat Cancer. 2022 Nov 21;3(11):1351–66. doi: 10.1038/s43018-022-00456-0 (PMC9701141; doi:10.1038/s43018-022-00456-0)
Supplement: Supplementary file 1 — Reporting Summary. [file 43018_2022_456_MOESM1_ESM.pdf]

nature portfolio

Corresponding author(s): Julien Sage

Last updated by author(s): 2022/9/28

Reporting Summary

Nature Portfolio wishes to improve the reproducibility of the work that we publish. This form provides structure for consistency and transparency in reporting. For further information on Nature Portfolio policies, see our Editorial Policies and the Editorial Policy Checklist.

Statistics

For all statistical analyses, confirm that the following items are present in the figure legend, table legend, main text, or Methods section.

n/a	Confirmed	
☐	☒	The exact sample size (*n*) for each experimental group/condition, given as a discrete number and unit of measurement
☐	☒	A statement on whether measurements were taken from distinct samples or whether the same sample was measured repeatedly
☐	☒	The statistical test(s) used AND whether they are one- or two-sided<br>*Only common tests should be described solely by name; describe more complex techniques in the Methods section.*
☐	☒	A description of all covariates tested
☐	☒	A description of any assumptions or corrections, such as tests of normality and adjustment for multiple comparisons
☐	☒	A full description of the statistical parameters including central tendency (e.g. means) or other basic estimates (e.g. regression coefficient) AND variation (e.g. standard deviation) or associated estimates of uncertainty (e.g. confidence intervals)
☐	☒	For null hypothesis testing, the test statistic (e.g. *F*, *t*, *r*) with confidence intervals, effect sizes, degrees of freedom and *P* value noted<br>*Give P values as exact values whenever suitable.*
☒	☐	For Bayesian analysis, information on the choice of priors and Markov chain Monte Carlo settings
☒	☐	For hierarchical and complex designs, identification of the appropriate level for tests and full reporting of outcomes
☒	☐	Estimates of effect sizes (e.g. Cohen's *d*, Pearson's *r*), indicating how they were calculated

Our web collection on statistics for biologists contains articles on many of the points above.

Software and code

Policy information about availability of computer code

Data collection	For RNA-seq, cell pellets were collected and sent to Novogene (en.novogene.com) for RNA extraction and Illumina sequencing. For CIBERSORT, processed data from GSE59733 and GSE15781 were downloaded from Gene Expression Omnibus (GEO). For signle-cell RNA seq, 5,000-10,000 cells per samples were barcoded and libraries were generated using the V2 10X Chromium system. The samples were aggregated and sequenced using NovaSeq with a target of 30,000 reads per cell. For a clinical SCLC case with abscopal effect, whole RNA was sequenced as previously described (Lissa D et al. Nat Commun. 2022 Apr 19;13(1):2023.)
Data analysis	For RNA-seq analysis, reads were quantified based on the mouse reference genome mm10 using Salmon using default settings. Differentially expressed genes were obtained using DESeq2 using IHW for p-value correction. Plots were generated ggplot2 (https://ggplot2.tidyverse.org). Genes were selected by filtering for log2 fold change > 1.5 or < -1.5 with corrected p-value < 0.05. GO pathway analysis was performed using Metascape (metascape.org). For CIBERSORT analysis, CIBERSORTx (https://cibersortx.stanford.edu/) was used to enumerate immune cell abundance in patient tumor samples that underwent transcriptomic profiling. For single-cell RNA-seq analysis, the scRNA-seq data from each sample were individually pre-processed using the Cellranger v6.0 pipeline. The downstream analysis on these datasets was performed using the Seurat v4 package. For a clinical SCLC case with abscopal effect, RNA sequencing was analyzed as previously described (Lissa D et al. Nat Commun. 2022 Apr 19;13(1):2023.)

For manuscripts utilizing custom algorithms or software that are central to the research but not yet described in published literature, software must be made available to editors and reviewers. We strongly encourage code deposition in a community repository (e.g. GitHub). See the Nature Portfolio guidelines for submitting code & software for further information.

Data

Policy information about availability of data

All manuscripts must include a data availability statement. This statement should provide the following information, where applicable:

- Accession codes, unique identifiers, or web links for publicly available datasets
- A description of any restrictions on data availability
- For clinical datasets or third party data, please ensure that the statement adheres to our policy

The accession number for the RNA-Seq results uploaded on the Gene Expression Omnibus database is GEO: 156106. All other data are available in the article and supplementary materials, or from the corresponding author upon reasonable request.

Human research participants

Policy information about studies involving human research participants and Sex and Gender in Research.

Reporting on sex and gender	The patient who achieved abscopal effect was female.
Population characteristics	The patient who achieved abscopal effect was initially treated with four cycles of carboplatin and etoposide. The patient had a recurrence in a left breast mass, mediastinal lymphadenopathy, liver lesions, and a pancreatic mass, 76 days after completion of the platinum-based chemotherapy. Subsequently the patient was enrolled in two investigational combination treatment clinical trials at National Cancer Institute. After palliative radiation to growing mediastinal and left breast lesions, the patient was treated on a clinical trial of M7824 (bintrafusp alfa, NCT03554473) and achieved abscopal responses. The patient was provided written informed consent. The trial was conducted under an institutional review board approval (NCI IRB identifier: 18-c-0110).
Recruitment	The patient was enrolled in a clinical trial of M7824 (bintrafusp alfa, NCT03554473) conducted at National Cancer Institute.
Ethics oversight	The patient was provided written informed consent. The trial was conducted under an institutional review board approval (NCI IRB identifier: 18-c-0110).

Note that full information on the approval of the study protocol must also be provided in the manuscript.

Field-specific reporting

Please select the one below that is the best fit for your research. If you are not sure, read the appropriate sections before making your selection.

☒ Life sciences ☐ Behavioural & social sciences ☐ Ecological, evolutionary & environmental sciences

For a reference copy of the document with all sections, see nature.com/documents/nr-reporting-summary-flat.pdf

Life sciences study design

All studies must disclose on these points even when the disclosure is negative.

Sample size	No statistical methods were used to predetermine sample size. For mouse studies, sample size was determined based on previous experience with the mouse model. For the majority of in vitro experiments, statistical analysis was performed on a minimum of three independent experiments.
Data exclusions	No data was excluded.
Replication	All in vitro experiments were performed at least twice on independent samples, with the majority of experiments performed 3 or more times All results were reproducible. Some of mouse studies were not replicated but included sufficient sample sizes.
Randomization	For all the in vivo experiments, the animals were randomly distributed and assigned to different treatment groups prior to the start of the treatment.
Blinding	Experiments reported in this study were not blinded.

Reporting for specific materials, systems and methods

We require information from authors about some types of materials, experimental systems and methods used in many studies. Here, indicate whether each material, system or method listed is relevant to your study. If you are not sure if a list item applies to your research, read the appropriate section before selecting a response.

Materials & experimental systems

n/a	Involved in the study	
☐	☒	Antibodies
☐	☒	Eukaryotic cell lines
☒	☐	Palaeontology and archaeology
☐	☒	Animals and other organisms
☐	☒	Clinical data
☒	☐	Dual use research of concern

Methods

n/a	Involved in the study	
☒	☐	ChIP-seq
☐	☒	Flow cytometry
☒	☐	MRI-based neuroimaging

Antibodies

Antibodies used

Brilliant Violet 421 anti-mouse CD45 antibody (1:200, 103133, Biolegend), PerCP/Cyanine5.5 anti-mouse CD3 antibody (1:200, 100327, Biolegend), Alexa Fluor 488 anti-mouse CD4 antibody (1:200, 100423, Biolegend), PE anti-mouse CD8a antibody (1:200, 100708, Biolegend), Brilliant Violet 785 anti-mouse/human CD11b antibody (1:200, 101243, Biolegend), PE anti-mouse F4/80 antibody (1:200, 123110, Biolegend), rat anti-mouse F4/80 antibody (1:50, 14480182, Invitrogen), Alexa Fluor 488 anti-mouse CD206 antibody (1:200, 141710, C068C2, Biolegend), APC anti-mouse CD11c antibody (1:200, 117310, N418, Biolegend), PE anti-mouse CD86 antibody (1:200, 105008, GL-1, Biolegend), anti-mouse CD47 antibody (MIAP410, Bio X Cell), anti-human CD47 antibody (B6H12, Bio X Cell), anti-CD8alpha antibody (2.43, Bio X Cell), anti-CSF-1 antibody (5A1, Bio X Cell), anti-ASCL1 (1:1000, #556604, BD Bioscience), anti-NeuroD1 (1:1000, #4373, Cell Signaling Technology), anti-c-MYC (1:1000, #5605, Cell Signaling Technology), anti-HSP90 (1:1000, #4877, Cell Signaling Technology)

Validation

All primary antibodies used in this study were validated by the vendors and/or are from well-known and characterized clones. Further details in validation can be found in Biolegend Reproducibility and Validation webpage (https://www.biolegend.com/en-us/reproducibility) or ThermoFisherScientific (https://www.thermofisher.com/us/en/home/lifescience/antibodies/invitrogen-antibody-validation.html).

Eukaryotic cell lines

Policy information about cell lines and Sex and Gender in Research

Cell line source(s)

NJH29 cells were described before (PMID: 24078773) and propagated in our laboratory. Rb/p53 mutant mouse SCLC KP1 and KP3 cells were previously described (PMID: 21983857) and propagated in our laboratory. Other cell lines (NCI-H82, NCI-H69, NCI-H526, Ramos, J774 cells) were from ATCC.

Authentication

None of the cell lines was authenticated.

Mycoplasma contamination

All cell lines tested negative for mycoplasma.

Commonly misidentified lines (See ICLAC register)

No commonly misidentified cell lines were used.

Animals and other research organisms

Policy information about studies involving animals; ARRIVE guidelines recommended for reporting animal research, and Sex and Gender in Research

Laboratory animals

Nod.Cg-PrkdcscidIL2rgtm1WjI/SzJ (NSG) mice (Jackson Laboratories, Stock No: 005557) were used for experiments in immunodeficient recipients. B6.129S F1 mice (Jackson Laboratories, Stock No: 101043) and C57BL/6J mice (Jackson Laboratories, Stock No: 000664) were used for experiments in immunocompetent recipients. Mice were engrafted with 0.5-2 million cancer cells in antibiotic-free serum-free media with 1:1 mixture of Matrigel (BD Matrigel, 356237) at 6-15 weeks of age. The tumors did not exceed the 1.75cm diameter permitted by our animal protocol.

Wild animals

This study did not involve wild animals.

Reporting on sex

The findings apply to both sexes. Both male and female mice were used in this study. No selection for sex of mice was performed.

Field-collected samples

This study did not involve field-collected samples.

Ethics oversight

Mice were maintained according to practices prescribed by the NIH and by the Institutional Animal Care and Use Committee (IACUC) at Stanford. Additional accreditation of the Stanford animal research facility was provided by the Association for Assessment and Accreditation of Laboratory Animal Care (AAALAC). The study protocol was approved by the Administrative Panel on Laboratory Animal Care (APLAC) at Stanford (protocol #APLAC-32397).

Note that full information on the approval of the study protocol must also be provided in the manuscript.

Clinical data

Policy information about clinical studies

All manuscripts should comply with the ICMJE guidelines for publication of clinical research and a completed CONSORT checklist must be included with all submissions.

Clinical trial registration	The patient was enrolled in a clinical trial of M7824 (bintrafusp alfa, NCT03554473) conducted at National Cancer Institute.
Study protocol	The full trial protocol can be accessed under ClinicalTrials.gov Identifier NCT03554473.
Data collection	The patient data was collected at National Cancer Institute.
Outcomes	Tumor samples of metastatic left cervical lymphadenopathy were collected pre- and post- radiation by experienced interventional radiologists at the National Institutes of Health for research purposes. Tumor RNA was sequenced and normalized to log2-transformed Trimmed mean of M values normalized fragments per kilobase of exon per million reads mapped as previously described. To deconvolute bulk gene expression data to immune subsets and SCLC transcriptomic subtype, we applied CIBERSORTx with default parameters. For immune subset analysis, we used the LMP6 gene set and weight.

Flow Cytometry

Plots

Confirm that:

☒ The axis labels state the marker and fluorochrome used (e.g. CD4-FITC).

☒ The axis scales are clearly visible. Include numbers along axes only for bottom left plot of group (a 'group' is an analysis of identical markers).

☒ All plots are contour plots with outliers or pseudocolor plots.

☒ A numerical value for number of cells or percentage (with statistics) is provided.

Methodology

Sample preparation	Flow cytometry experiments were performed on cell lines and tumor samples. For in vitro experiments, cells were collected, washed in PBS and stained with antibodies according to standard procedures. For in vivo experiments, to create cell suspensions, tumors were removed, finely chopped, and suspended in PBS. Tumors were digested with collagenase/Dispase for 30min at 37ºC then filtered through a 40µm mesh. Cells were resuspended in red blood cell lysis buffer for 1min at room temperature. Cells were resuspended in PBS, counted, Fc receptors were blocked with CD16/32 Ab (BioLegend), and then 1 million cells were stained with conjugated Ab cocktail for 20min on ice. Cells were washed two times in PBS, and then resuspended for flow cytometry analysis.
Instrument	Flow cytometry analysis was performed on either a BD LSRFortessa, a BD FACSAria II (BD Biosciences).
Software	Data were collected using either BD FACSDiva software (BD Biosciences). Data were analyzed using FlowJo v10.
Cell population abundance	CD45+ viable cells consisted of nearly 98% of the population of cells collected per sample.
Gating strategy	For all experiments, all cells were gated by FSC area vs. SSC area, and singlets were gated by SSC height vs SSC width. From here, these were further gated on CD45+CD11b+F4/80+ for macrophages analysis. Fluorophores were chosen to minimize spectral overlap. Compensation beads labeled with appropriate antibodies were used prior to all data collection (UltraComp eBeads 01-2222-42 eBiosciences).

☒ Tick this box to confirm that a figure exemplifying the gating strategy is provided in the Supplementary Information.
